# Sensing of SARS-CoV-2-infected cells by plasmacytoid dendritic cells is modulated via an interplay between CD54/ICAM-1 and CD11a/LFA-1 α_L_ integrin

**DOI:** 10.1128/jvi.01235-24

**Published:** 2025-01-13

**Authors:** ChenRongRong Cai, Tram N. Q. Pham, Damien Adam, Emmanuelle Brochiero, Éric A. Cohen

**Affiliations:** 1 Institut de recherches cliniques de Montréal, Montréal, Québec, Canada; 2 Département de microbiologie, infectiologie et immunologie, Faculté de médecine, Université de Montréal 5622 , Montréal, Québec, Canada; 3 Centre de recherche du Centre Hospitalier de l'Université de Montréal, Centre de recherche du Centre Hospitalier de l'Université de Montréal 177460 , Montréal, Québec, Canada; 4 Département de médecine, Université de Montréal 5622 , Montréal, Québec, Canada; The Ohio State University, Columbus, Ohio, USA

**Keywords:** SARS-CoV-2 VOCs, plasmacytoid dendritic cells, IFN-I, conjugate formation, CD54, CD11a, innate sensing

## Abstract

SARS-CoV-2 infection induces interferon (IFN) response by plasmacytoid dendritic cells (pDCs), but the underlying mechanisms are poorly defined. Here, we show that the bulk of the IFN-I release comes from pDC sensing of infected cells and not cell-free virions. Physical contact (or conjugates) between pDCs and infected cells is mediated through CD54-CD11a engagement, and such conjugate formation is required for efficient IFN-I production. Interestingly, CD11a is inducible on infected epithelial cells when they are co-cultured with PBMCs, thus allowing for potentially bidirectional cross-talks between CD54 and CD11a, which further amplify the sensing. SARS-CoV-2 variants of concern (VOCs) are sensed less efficiently than the Wuhan ancestral strain (LSPQ1), but the mechanisms driving the defect are different among the VOCs. CD11a induction on infected cells is correlated with their ability to form cell conjugates with pDCs. Impaired sensing of the Alpha variant is linked to reduced CD11a induction on infected cells and to fewer conjugates formed with pDCs. Collectively, our findings provide new insights into how SARS-CoV-2-infected cells are sensed by pDCs and reveal that this process is targeted by some VOCs to limit IFN-I production.

**IMPORTANCE:**

Type I interferons (IFN-I) represent an important component of the host’s innate defense against initial SARS-CoV-2 infections. Plasmacytoid dendritic cells (pDCs) produce large quantities of IFN-I upon recognition of viral particles or infected cells. This study shows that pDCs sense infected cells more efficiently than viral particles, leading to a higher production of IFN-I. Physical contact between a pDC and an infected cell is critical to this process; the interaction is mediated via CD11a and ICAM-1 complex and potentially is bidirectional. SARS-CoV-2 variants of concern (VOCs) have evolved to limit the IFN response through different mechanisms. For the Alpha variant, reduced level of CD11a on infected cells is linked to less contact with pDCs and decreased IFN-I release. Overall, our study characterizes some of the early steps involved in pDC-mediated response against SARS-CoV-2 infection and shows that these processes are targeted by VOCs to likely limit IFN-I response and enhance viral spread.

## INTRODUCTION

Severe acute respiratory syndrome coronavirus 2 (SARS-CoV-2) is the etiological agent of coronavirus disease 19 (COVID-19) (1), which has caused millions of deaths worldwide. Patients with severe or life-threatening COVID-19 have been shown, in some studies, to have impaired type I interferon (IFN-I) responses (2–6). Some of these individuals are genetically unable to make IFN-I, whereas others develop autoantibodies against IFN-α (5, 6). Collectively, these findings highlight the protective role of IFN-I in SARS-CoV-2 infection. However, IFN-I can be a double-edged sword because its elevated and prolonged production is correlated with inflammation, an observation seen only in patients with severe COVID-19 (7). Consequently, understanding how detection of SARS-CoV-2 infection triggers IFN-I production and how the virus might evolve to interfere with this process can further our knowledge of the pathogenesis of SARS-CoV-2 infection. In this context, different SARS-CoV-2 variants of concern (VOCs) have acquired adaptations to infect humans more efficiently, partly through evading innate immune responses (8, 9). Indeed, SARS-CoV-2 variants that emerged later during the COVID-19 pandemic, such as the Omicron, possess an enhanced ability to resist the antiviral effect of IFNs compared with the earlier strains. One example is the Omicron BA.1 variant, which is highly resistant to IFN-I treatment (10).

Plasmacytoid dendritic cells (pDCs) are a distinct subset of DCs that release IFNs upon activation through RNA- and DNA-sensing via endosomal toll-like receptors (TLRs) 7 and 9, respectively (11). They are major producers of IFNs and, as such, act not only as the first line of host defense against invading pathogens but also as a bridge between the innate and adaptive arms of immunity. Although pDCs are reported to sense SARS-CoV-2 virions (12–14), there is increasing interest in examining to what degree they sense infected cells (2). Understanding the molecular mechanism(s) underlying this mode of sensing is particularly important to uncover whether SARS-CoV-2 interferes with pDC sensing to limit IFN-I antiviral responses as in the case of other viruses (15–17). Furthermore, whether cells infected with VOCs are differentially sensed by pDCs remains to be established.

Recognition of infected cells by pDCs usually relies on the ability of pDCs to form physical contact with infected cells (16–18). This process, which is mediated by cell membrane receptors and adhesion molecules, such as intercellular adhesion molecule-1 (ICAM-1 and CD54) and its ligand, CD11a/CD18 (α_L_β_2_ integrin, LFA-1), leads to the formation of conjugates or interferogenic synapses (2, 16, 18).

In this study, we investigated the molecular mechanisms underlying the sensing of SARS-CoV-2-infected cells by pDCs using different human epithelial cell models. We confirm that pDCs are the main producers of IFN-I in SARS-CoV-2 infection and establish that pDCs sense infected cells much more efficiently than they do cell-free viruses. PDCs make direct physical contact with infected cells through CD54 (ICAM-1) and its ligand CD11a (LFA-1 α_L_ integrin) interactions, and formation of such cell conjugates is needed to drive IFN-I production. Interestingly, we provide evidence that CD11a expression on infected cells could be induced when they are in co-culture with PBMCs and that CD54-CD11a interactions might be bidirectional. Importantly, we document that human epithelial cells infected with the Alpha, Delta, or Omicron variants are sensed by pDCs significantly less efficiently compared with a near Wuhan ancestral strain (LSPQ1), and the mechanism(s) driving the defect appear to be different among the VOCs. We uncover that formation of cell conjugates with pDCs is correlated with the extent of CD11a induction. For the Alpha variant, this suboptimal sensing is linked to a lower level of CD11a induced on infected cells and fewer conjugates formed with pDCs. Taken together, our findings reveal key information about the mechanism by which pDCs recognize and sense SARS-CoV-2 infected cells and highlight how the virus has evolved to interfere with these processes to limit IFN-I production.

## RESULTS

### Sensing of SARS-CoV-2-infected cells by pDCs is more efficient compared with cell-free virions

**Fig 1:**
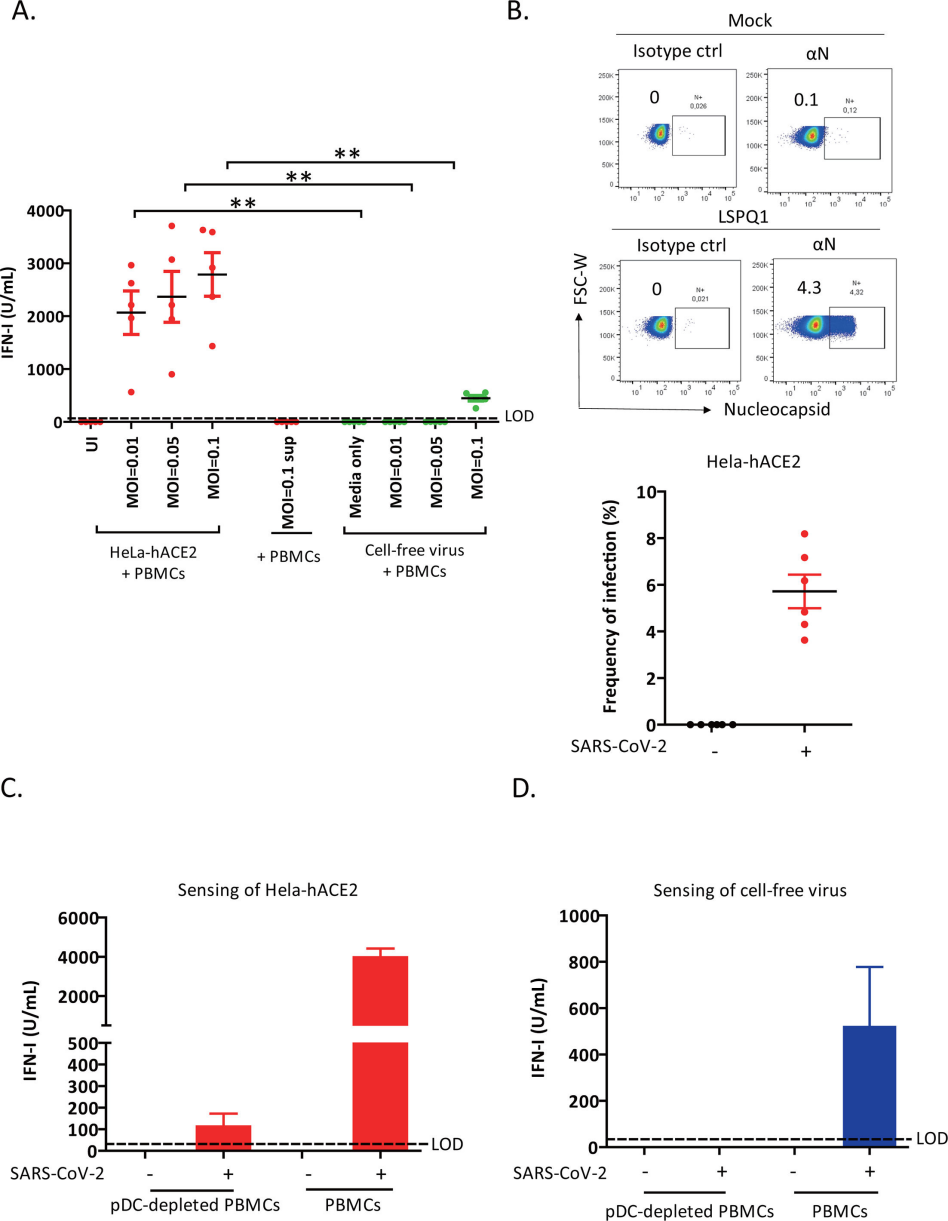
Sensing of SARS-CoV-2-infected cells by pDCs is more efficient compared with cell-free virions. (**A**) Quantification of IFN-I (IFN-α/β) in the supernatant of PBMCs that were co-cultured for 24 h with SARS-CoV-2 (LSPQ1)-infected HeLa-hACE2, exposed to cell-free SARS-CoV-2 at different MOIs, or treated with supernatant of infected cells (MOI 0.1) (sup) collected at 24 h post-infection. PBMCs used in the co-cultures and in “cell-free virus exposure” conditions were from the same donors with the same number of cells. In the latter conditions, the MOI was based on the number of PBMCs. UI, uninfected cells. Each dot represents independent experiments/donors. Mann-Whitney *U* test. **P < 0.01. (**B**) (Upper panel) Frequency of SARS-CoV-2 infection in HeLa-hACE2 as determined by flow cytometry using αN Ab and a mouse IgG1 isotype control (ctrl). (Lower panel) A summary dot plot shows the results from six independent infections. (**C**) Quantification of IFN-I in the supernatant of SARS-CoV-2-infected HeLa-hACE2 (MOI 0.1, 24 h) that were co-cultured with pDC-depleted PBMCs or total PBMCs (*n* = 3 donors). (**D**) The analysis was as described for panel C but with cell-free viruses instead of infected cells (*n* = 2 donors). The MOI (0.1) used was based on the number of PBMCs, as done in Fig. 1A. In all relevant panels, the co-cultures were done for 24 h. Shown is the mean ± SEM. The dotted line indicates the limit of detection (LOD) of the assay (60 U/mL). See also Fig. S1.

Sensing of cell-free SARS-CoV-2 particles has previously been reported (12–14), but knowledge concerning sensing of infected cells is incomplete (2). Thus, we asked whether pDCs could sense infected cells and whether the magnitude of pDC response differs between these two modes of sensing SARS-CoV-2 infection. To this end, PBMCs from healthy human donors were co-cultured with HeLa-hACE2 that had been infected for 24 h with different multiplicities of infection (MOIs) of Wuhan-like LSPQ1 strain. Alternatively, the PBMCs were exposed for up to 24 h to either LSPQ1 cell-free virions at the same MOI used to infect HeLa-hACE2 or to the culture media harvested from the infected HeLa-hACE2 cell cultures (MOI 0.1). The resulting supernatant was analyzed for bioactive IFN-I production using a HEK-Blue IFN-α/β based reporter assay. Since IFN-I was detectable starting at 18 h after treatment with PBMCs (Fig. S1A), we chose the 24 h time point to assess for IFN-I production in subsequent experiments. Interestingly, we find that sensing of infected cells could be observed starting at MOI of 0.001, whereas that of cell-free virions was evident only at an input corresponding to a MOI of 0.1 (Fig. 1A). In fact, at this MOI, the IFN-I production released upon sensing of infected cells was approximately 6-fold higher compared with that of cell-free virions. Unsurprisingly, no IFN-I was detectable when uninfected cells were co-cultured with PBMCs (Fig. 1A). It was evident that at 24 h post-infection, infected cells did not produce detectable levels of IFN-I, and the supernatant from the 24 h infected cultures did not induce IFN-I production when added to PBMCs (MOI 0.1 sup) (Fig. 1A; Fig. S1B). It was only at 48 h post-infection that IFN-I became detectable in the supernatant of infected cultures (Fig. S1B). We observe that only a small fraction of infected cells, as determined by flow cytometry using anti-SARS-CoV-2 nucleocapsid (ɑN) antibodies (Ab), was needed to drive the IFN-I release (Fig. 1B). The lack of staining with an isotype control validates the authenticity of the fluorescent signal observed with the αN Ab (Fig. 1B). Depleting pDCs from PBMCs essentially prevented IFN-I production (Fig. 1C and D), indicating that pDCs are a major source of IFN-I upon recognizing infected cells (Fig. 1C) or cell-free virus particles (Fig. 1D). In this context of pDC sensing of infected cells, our data are consistent with that of an earlier report (2). We find that no human angiotensin-converting enzyme 2 (hACE2), the main SARS-CoV-2 entry receptor (1), was detectable at the surface of BDCA2^+^ILT7^+^ pDCs (Fig. S1C and D), suggesting that IFN-I production is unlikely to be a consequence of viral entry through the hACE2 receptor.

We next quantified the level of virions in infected HeLa-hACE2 to assess whether the enhanced sensing of infected cells compared with that of cell-free virions (Fig. 1) could simply be due to infected cells presenting more viral particles to pDCs. To this end, we found that at 4%–8% infection (Fig. 1B), the number of infectious SARS-CoV-2 virions in infected cells was 1,600-fold lower than that in the viral inoculum used to stimulate PBMCs. However, infected cells were a more potent stimulator compared with cell-free viral particles (Fig. S1E). Collectively, our data demonstrate that pDCs sense SARS-CoV-2-infected cells more efficiently than they do cell-free virions and that the enhanced sensing cannot be attributed to the sheer number of viral particles on and in infected cells.

### PDC sensing of SARS-CoV-2-infected cells requires physical contact

**Fig 2:**
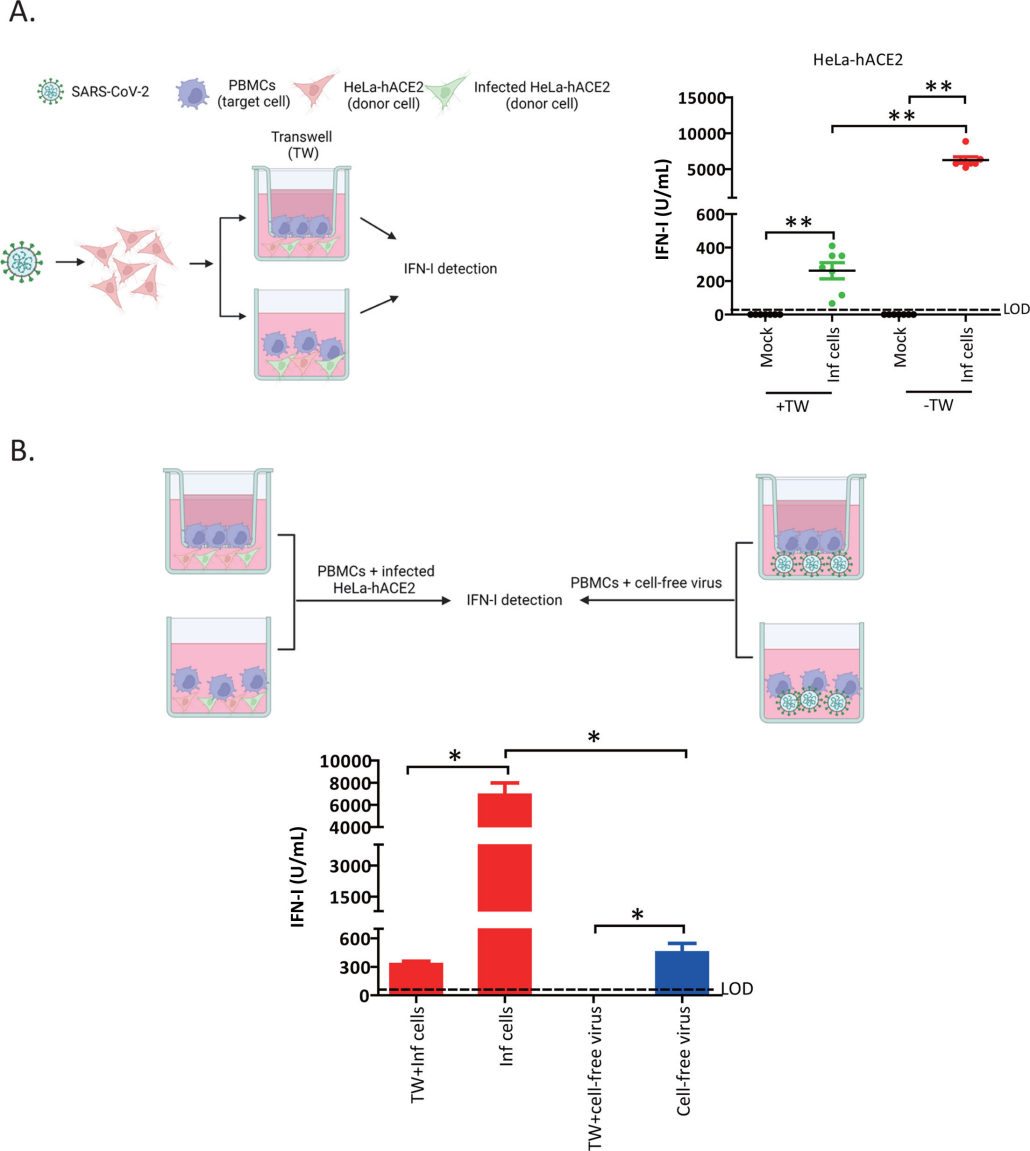
pDC sensing of SARS-CoV-2-infected cells requires physical contact. (**A**) (Left panel) Schematic representation of the experimental design (created with BioRender.com). HeLa-hACE2 cells were infected with SARS-CoV-2 at MOI of 0.1 for 24 h and then co-cultured with PBMCs in the presence or absence of a 0.4 µm transwell membrane insert. PBMCs were added to the upper chamber of the insert. IFN-I was measured from the lower chamber, at 18–24 h after the co-cultures. (Right panel) Quantification of IFN-I in the supernatant of PBMCs co-cultured with mock- or SARS-CoV-2-infected HeLa-hACE2 (Inf cells) in the presence (+TW) or absence (–TW) of a transwell insert. Inf cells, infected cells. Data shown are mean ± SEM from independent experiments with seven donors. Each dot depicts a donor. Mann-Whitney *U* test. **P < 0.01. (**B**) (Upper panel) Design of an experiment aimed at determining whether cell-free virus can trigger residual IFN-I production in the absence of physical contact (created with BioRender.com). (Lower panel) IFN-I was measured in supernatants of PBMCs either co-cultured with infected HeLa-hACE2 (MOI 0.1) in the presence or absence of transwell inserts or exposed to cell-free virus (MOI 0.1, based on PBMC number) in the same conditions. The number of PBMCs used for the co-cultures or exposed to cell-free viruses was the same. Data shown are mean ± SEM from independent experiments with four donors. Mann-Whitney *U* test. *P < 0.05. In both panels: LOD, limit of detection of the IFN-I assay as described in Fig. 1A legend. See also Fig. S1.

Given that pDC-mediated responses to viral infections usually require physical contact with infected cells (16–18), we asked whether direct contact between pDCs and SARS-CoV-2-infected cells was necessary for efficient IFN-I production. Thus, co-culture experiments were performed under the condition where pDC-containing PBMCs were separated from infected cells using a 0.4 µm transwell membrane insert (+TW condition, Fig. 2, left panel). Here, we observed a 90%–95% attenuation in IFN-I production in the presence of the membrane insert (Fig. 2A, right panel), indicating that physical contact between SARS-CoV-2-infected cells and pDCs was critical for sensing and eliciting IFN-I production.

We next assessed whether the residual IFN-I release (Fig. 2A, green dots) was due to sensing of cell-free virions that had been liberated from infected cells and passed through the transwell inserts. For this purpose, cell-free virus was exposed to PBMCs in the presence or absence of the transwell filters. As shown in Fig. 2B, we observed a complete loss of IFN-I release (Fig. 2B, compare TW + cell-free virus with cell-free virus conditions), supporting the notion that the observed residual IFN-I release was not a consequence of pDCs sensing viral particles produced by infected cells (Fig. 2B). In fact, the data point toward infected cells being the likely source of the residual IFN-I release (Fig. 2B, compare TW + infected cells with TW + cell-free virus conditions), and this is indeed consistent with the finding that infected cells release a modest level of IFN-I at 48 h post-infection (Fig. S1B). Altogether, our data corroborate previous data (2) in demonstrating that physical contact between pDCs and infected cells is a prerequisite for efficient sensing of SARS-CoV-2. In addition, we uncover that virus particles transmitted via cell-to-cell contact are more potent inducers of IFN-I from pDCs than cell-free viruses.

### PDCs can sense SARS-CoV-2-infected polarized cell lines and differentiated primary human airway epithelial cells from the basolateral domain

**Fig 3:**
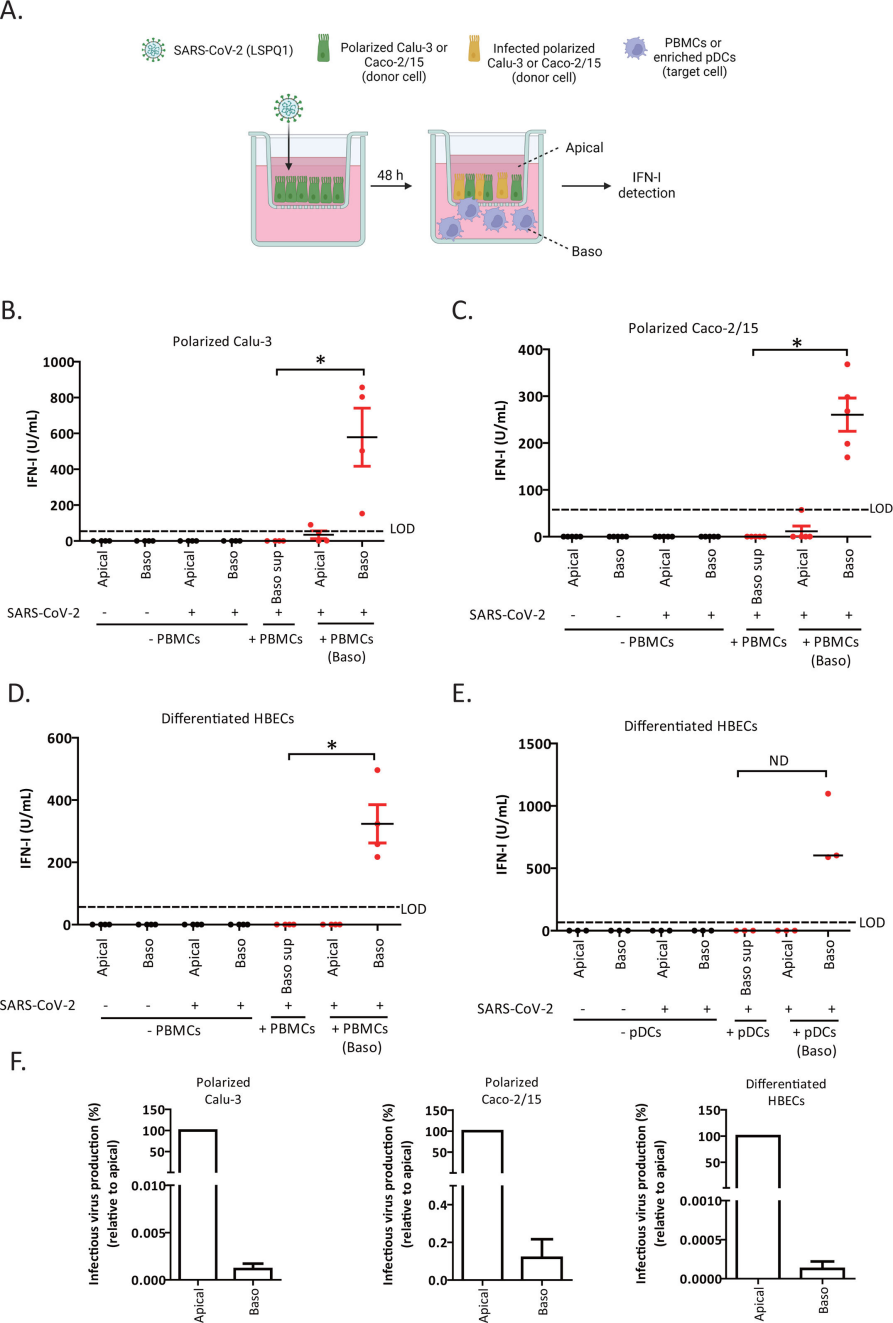
pDCs can sense SARS-CoV-2-infected polarized epithelial cell lines and differentiated primary human airways cells from the basolateral domain. (**A**) Design of a sensing experiment with polarized cells (created with BioRender.com). Calu-3 or Caco-2/15 were infected for 48 h with SARS-CoV-2 (MOI 0.1). PBMCs or enriched pDCs were added to the lower chamber facing the basolateral domain of the polarized Calu-3 or Caco-2/15 cells to mimic an *in vivo* condition. IFN-I was measured in both chambers 18–24 h later. (**B–D**) Quantification of IFN-I in supernatants of mock- and SARS-CoV-2-infected cells in the absence (black dots) or presence (red dots) of PBMCs added to the basolateral side. Calu-3 (**B**), Caco-2/15 (**C**), and primary human bronchial epithelial cell (HBEC) (**D**) were polarized/differentiated on a 3.0 µm pore size transwell insert and infected with SARS-CoV-2 from the apical domain. Each black dot represents supernatant from independent experiments (no PBMC co-culture). Red dots (apical and baso) depict supernatant obtained following co-cultures of infected cells with PBMCs. Each dot is an analysis with a distinct donor. Alternatively, the supernatants collected from the basolateral side of infected cells (Baso sup) were exposed to the same donors used in the co-culture conditions. Apical and baso, apical and basolateral sides of a transwell insert, respectively. Data are shown as mean ± SEM from independent experiments with four or five donors. Mann-Whitney *U* test. *P < 0.05, **P < 0.01. (**E**) Quantification of IFN-I in supernatants of mock- and SARS-CoV-2-infected differentiated HBECs in the presence or absence of pDCs added to the basolateral side of the transwell. Each black dot represents an HBEC culture with a different donor (*n* = 3). Each red dot depicts a pDC donor either co-cultured with HBECs (Baso) or exposed to the supernatant collected from the basolateral side of the infected HBEC culture (Baso sup) (*n* = 3). The horizontal line depicts the median. No statistical analysis could be performed due to the fact that we had only three donors, ND (not determined). (**F**) Directional release of SARS-CoV-2 infectious virus from the indicated polarized/differentiated cell types, as determined by a plaque assay. Release from the apical side was set as 100% and that from the basolateral side was expressed as the percentage of the respective apical release. Calu-3 (*n* = 3), Caco-2/15 (*n* = 2), and HBECs (*n* = 3). Data are shown as mean ± SEM. See also Fig. S2.

Airway epithelial cells, the primary gateway for SARS-CoV-2 infection, are organized in a polarized/differentiated fashion with distinct apical and basolateral domains *in vivo* (19). Although the apical domain is exposed to the outside lumen and external environment, the basolateral domain is in contact with neighboring cells and the basal lamina, which contains immune cells such as pDCs (20). Increasing evidence has demonstrated that intestinal epithelial cells, which are also organized in a polarized fashion, represent a potent alternative entry route for SARS-CoV-2 (21). Having demonstrated that pDCs can sense unpolarized infected model cells, we next assessed if the observation could be extended to polarized cell systems, given that in these physiologically relevant conditions, the bulk of SARS-CoV-1 release is reported to occur from the apical domain (22). Thus, human respiratory lung Calu-3 cells and intestinal Caco-2/15 cells were polarized on 3.0 µm transwell membrane inserts, which allow for cell migration and direct contact between cells across the membrane (23) and subsequently infected with SARS-CoV-2 from the apical domain (Fig. 3A). We found that no IFN-I was detectable in the supernatant obtained from both the apical (Apical) and basolateral (Baso) sides of uninfected or infected cells (Fig. 3B and C, black circles, ± SARS-CoV-2), suggesting that under these experimental conditions, quantifiable IFN-I was not released from infected epithelial cells. However, when Calu-3 or Caco-2/15 cells were co-cultured with PBMCs placed on the basolateral (Baso) side to mimic the microenvironment *in vivo*, IFN-I was readily detectable in the media harvested from the basolateral (Baso, red dots) but not apical side (Apical, red dots) (Fig. 3B and C). Also, no IFN-I was released when PBMCs were directly exposed to the viral supernatant from infected cultures (Baso sup, red dots), further confirming that the measurable IFN-I came from PBMC sensing of infected cells (Fig. 3B and C). Importantly, these results were reproducible in differentiated primary human bronchial epithelial cells (HBECs), which were co-cultured with PBMCs or isolated pDCs (Fig. 3D and E).

SARS-CoV-2 was predominantly released from the apical domain, and we observed this in all three cell types examined. (Fig. 3F). Interestingly, the presence of PBMCs did not affect the direction from which infectious virions were secreted: the bulk of the virus was still found at the apical domain (Fig. S2A). Despite that SARS-CoV-2 was predominantly detected in the supernatant collected from the apical side (Fig. 3F), no IFN-I was detectable from the apical or the basolateral sides in the absence of co-culture with PBMCs (Fig. 3B through E). Taken together, the data further strengthen our assertion that sensing of infected cells is key to pDC-mediated IFN-I production.

Of significance, although cells polarized on a 0.4 µm transwell insert were as susceptible to SARS-CoV-2 infection as those differentiated on a 3.0 µm membrane (Fig. S2B), IFN-I was only detected upon sensing of Caco-2/15 cells polarized on a 3.0 µm membrane (Fig. S2C). Similarly, when HBECs were differentiated on a 0.4 µm membrane insert, sensing of infected cells was highly impaired with detectable IFN-I observed with only one out of three PBMC donors tested, and the level was significantly lower (Fig. S2D) than that obtained from sensing of infected HBECs polarized on a 3.0 µm membrane (Fig. 3D). The data thus suggest that insufficient physical contact between pDCs and infected cells likely contributed to the impaired response. Indeed, Ma et al. (24) showed that 0.4 µm pores can only provide end-feet contact for small cell types such as astrocytes, which are smaller than epithelial cells (25) and limit the body to the other side of the membrane. Collectively, our data indicate that physical contact between pDCs and the basolateral domain of SARS-CoV-2-infected cells triggers efficient sensing regardless of the experimental cell systems. Additionally, the directional release of SARS-COV-2 occurs primarily from the apical domain regardless of whether PBMCs are present.

### CD11a expression is inducible on lung epithelial cells during co-cultures with PBMCs

**Fig 4:**
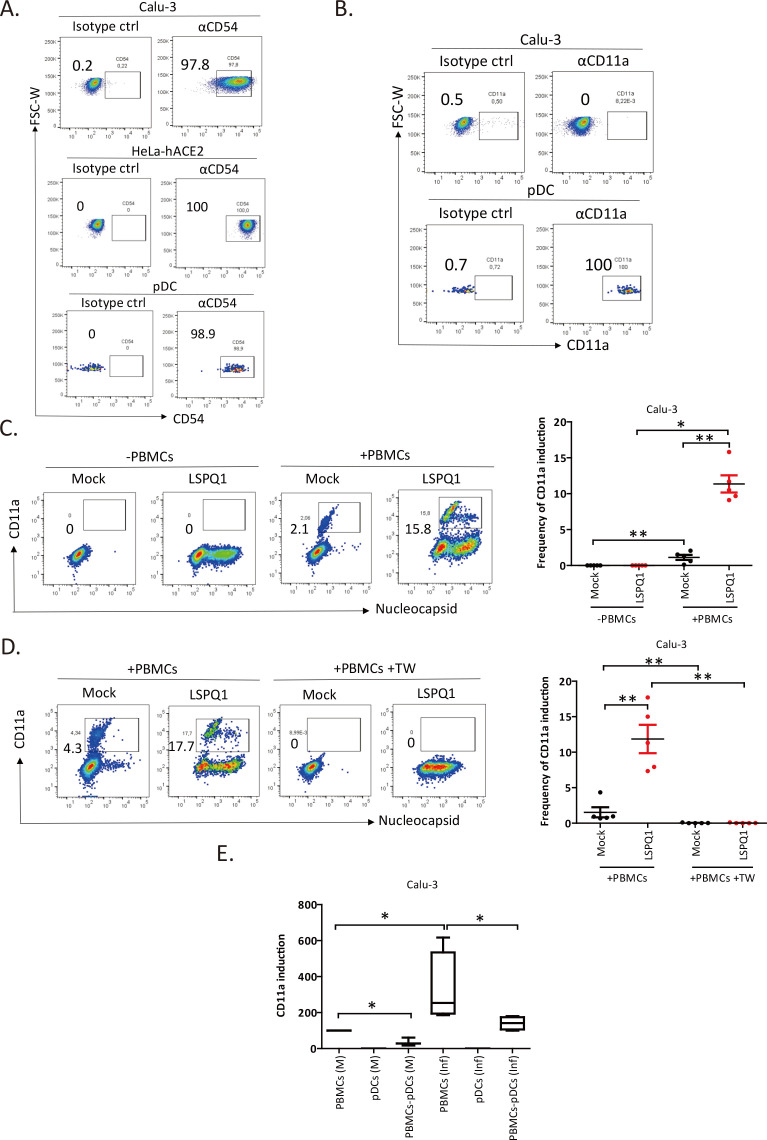
CD11a expression is inducible on lung epithelial cells during co-cultures with PBMCs. (**A and B**) Representative dot plots showing the frequency of cells expressing cell-surface CD54 and CD11a. Parallel staining with isotype controls instead of αCD54 or αCD11 Abs was done to confirm staining specificity. The numbers shown are the percentage of positive cells. (**C**) (Left panel) A representative dot plot showing the frequency of CD11a^+^ cells following a 24 h co-culture with or without PBMCs. Cells had been infected for 24 h at the time of the co-culture. (Right panel) Frequencies of CD11a^+^ cells obtained from independent co-cultures with five different PBMC donors. Each dot indicates a distinct PBMC donor. Compiled data are shown as mean ± SEM. Mann-Whitney *U* test. *P < 0.05, **P < 0.01. (**D**) (Left panel) Representative dot plots showing the frequency of CD11a^+^ cells in mock and infected cells co-cultured with PBMCs in the absence or presence of a 0.4 µm transwell insert. The experimental design is as described in Panel C legend. (Right panel) Compiled data from independent experiments (with five donors) are shown as mean ± SEM. Each dot depicts a different PBMC donor. Mann-Whitney *U* tests, **P < 0.01. (**E**) CD11a induction on Calu-3 after co-cultures with total PBMCs, enriched pDCs, or pDC-depleted PBMCs. Cells were mock-infected (M) or infected with the LSPQ1 SARS-CoV-2 (Inf) for 24 h at the start of the 24 h co-culture. CD11a expression on mock-infected Calu-3 after the co-culture with total PBMCs was set at 100%. Compiled data are shown in Whisker plots with median, minimum, and maximum values indicated. Total PBMCs and pDC-depleted PBMCs (*n* = 3), enriched pDCs (*n* = 2). Mann-Whitney *U* test. *P < 0.05. See also Fig. S3.

The establishment of physical contact requires cell adhesion complexes, which are composed of adhesion receptor(s) on the cell membrane that non-covalently bind to their ligands in neighboring cells or the extracellular matrix (26). CD54 known as the intercellular adhesion molecule-1 (ICAM-1) and its ligand CD11a, lymphocyte function associate antigen-1α (LFA-1α, or α_L_ integrin) have been shown to be important in the formation of physical contact between pDCs and virally infected cells (2, 18). Thus, to gain a better understanding of the mechanism by which CD54 and CD11a are involved in the context of pDCs making contact with SARS-CoV-2 infected cells, we first determined the expression of CD54 and CD11a on pDCs, HeLa-hACE2 and Calu-3. As shown, CD54 was highly expressed at the cell surface of HeLa-hACE2, Calu-3, and pDCs with nearly all cells expressing CD54 (Fig. 4A). However, CD11a was present only on pDCs but not Calu-3, with virtually no CD11a detectable on the latter when compared with that of the isotype control (Fig. 4B). Moreover, SARS-CoV-2 infection induced negligible expression on these cells (Fig. 4C, –PBMCs).

Given that environmental stimuli from co-cultures or cell-to-cell interactions can affect cell expression profile and activity (27), we assessed whether co-culturing PBMCs with infected Calu-3 cells could induce CD11a expression on the latter. To this end, we observed an average increase of 2.5% in the level of CD11a on mock-infected Calu-3 (Fig. S3A; Fig. 4C). However, the induction was significantly more pronounced when infected cells were co-cultured with PBMCs (approximately 6-fold to 8-fold higher than that given by mock-infected cells) (Fig. 4C). A parallel staining of mock and infected cells with an isotype control for αCD11a Ab confirmed the validity of the results (Fig. S3A).

It should be noted that PBMCs, which contain mostly non-adherent cells, were routinely removed at the end of the co-culture before adherent Calu-3 were detached by PBS/EDTA for flow cytometry. Nevertheless, in order to fully exclude the possibility that residual PBMCs might have contributed to the CD11a detection on Calu-3, a complementary analysis was done where, in addition to CD11a, cells were also simultaneously stained for CD3, CD14, CD19, HLA-DR, CD11c, BDCA2, and ILT7. As shown in Fig. S3B, gating out PBMCs (top two panels) did not significantly alter the level of CD11a expression on Calu-3 compared with the condition where PBMCs were not removed from the analysis (bottom two panels), suggesting that the majority of CD11a detected is mainly on Calu-3. Interestingly, contact between PBMCs and infected cells was critical since the use of 0.4 µm transwell inserts completely abolished CD11a induction on epithelial cells (Fig. 4D).

Since pDCs were responsible for the bulk of IFN-I production upon sensing infected cells (Fig. 1C and D) and co-culturing of infected cells with PBMCs induced CD11a expression (Fig. 4C and D), we next determined whether pDCs were involved in the augmentation of the integrin. To this end, we found that pDCs alone failed to effectively induce CD11a expression on either mock or infected Calu-3 cells (Fig. 4E). However, in both uninfected and infected cells, depletion of pDCs from PBMCs led to a statistically significant decrease in the level of CD11a induction, suggesting that in the presence of other cell subtypes within PBMCs, pDCs contribute to the induction of CD11a on Calu-3 (Fig. 4E). Interestingly, CD14^+^ monocytes alone moderately induced CD11a expression with the induction being more pronounced on infected cells (Fig. S3C). Accordingly, depletion of monocytes from PBMCs statistically decreased CD11a induction on both mock and infected cells (Fig. S3C), supporting a role for monocytes in the modulation of CD11a expression on epithelial cells. The observation that pDCs alone were unable to elicit CD11a expression, but their depletion from PBMCs led to a meaningful reduction in CD11a induction raises the possibility that monocytes might trigger CD11a expression on infected cells more efficiently in the context of pDC and infected cell conjugates (Fig. 4E; Fig. S3C). Similar analysis with CD4^+^ T cells (Fig. S3D) and CD19^+^ B cells (Fig. S3E) revealed no noticeable changes in the frequency of CD11a-positive cells, suggesting that these cell subtypes do not play a significant role in this process. Taken together, our data demonstrate that in an environment where different cell subsets in PBMCs can interact, CD11a expression could be stimulated on infected epithelial cells, a condition that could potentially strengthen contacts with pDCs via bidirectional and/or temporal interactions between CD54 and CD11a.

### CD54/CD11a adhesion complex is involved in the recognition and sensing of SARS-CoV-2-infected cells by pDCs in a potentially bidirectional manner

**Fig 5:**
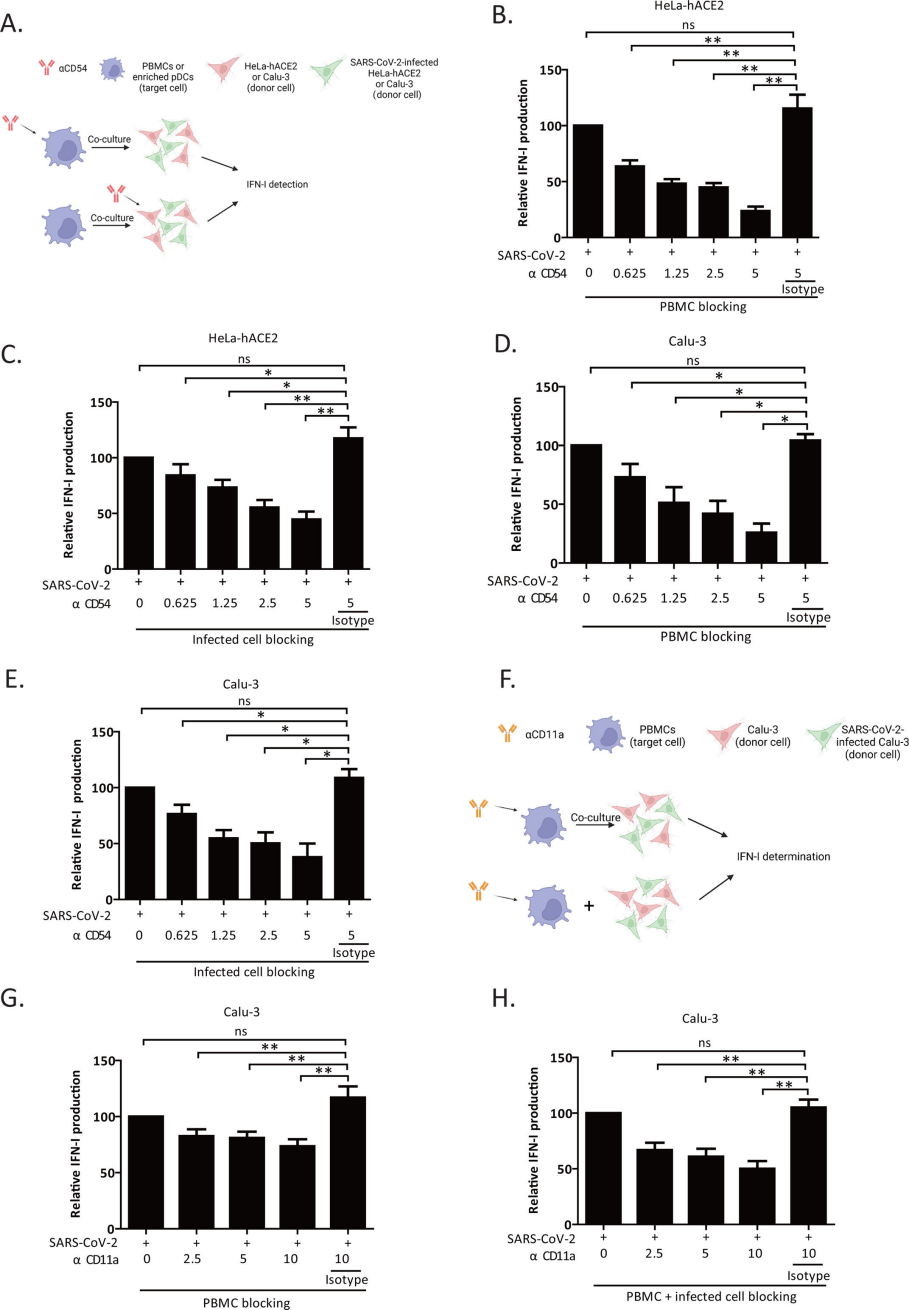
CD54/CD11a adhesion complex is involved in the recognition and sensing of SARS-CoV-2 infected cells by pDCs in a potentially bidirectional manner (**A**) Design of a CD54 blocking experiment (created with BioRender.com). PBMCs, enriched pDCs, or SARS-CoV-2-infected cells were pretreated with various concentrations of αCD54 Ab for 30 min at room temperature before the 24 h co-cultures. Cells treated with an isotype Ab control served as a negative control. (**B**) IFN-I in the supernatant of a co-culture between infected HeLa-hACE2 and αCD54 Ab-pretreated PBMCs. The IFN-I level in the Ab-untreated condition was set at 100%, six donors. (**C**) IFN-I production in the supernatant of a 24 h co-culture whereby infected HeLa-hACE2 were pretreated with αCD54 Ab and then exposed to PBMCs. The IFN-I level in the untreated condition was set at 100%, six donors. (**D**) IFN-I release from the supernatant of SARS-CoV-2-infected Calu-3 co-cultured with anti-CD54 Ab-pretreated PBMCs. Data analysis was as described for panel B, four donors. (**E**) IFN-I from a co-culture supernatant of PBMCs with infected Calu-3 that were treated with αCD54 Ab. Data analysis was as described for Panel B, four donors. (**F**) Experimental design of a CD11a blocking experiment (created with BioRender.com). Either PBMCs were pretreated with increasing concentrations of αCD11a Ab or the αCD11a Ab was added to a co-culture of PBMCs and infected Calu-3. IFN-I was determined 18–24 h post-co-culture. (**G and H**) IFN-I production in the supernatant of infected-Calu-3 co-cultured with PBMCs. (**G**) PBMCs were pretreated with the indicated concentrations of αCD11a Ab or isotype Ab control, and IFN-I release from a co-culture with untreated PBMCs was set at 100%. (**H**) αCD11a Ab was added to the co-culture of PBMCs and infected Calu-3. IFN-I production from the Ab-untreated co-culture was set as 100%, six donors. In all relevant panels, data are shown as mean ± SEM. Statistical analysis: Mann-Whitney *U* test; *P < 0.05, **P < 0.01, ns, not significant. See also Fig. S4.

Our data indicate that CD54 is expressed on infected donor cells and pDCs. The findings also show that its ligand CD11a is abundantly present on pDCs and can be induced on infected cells upon physical contact with PBMCs. Thus, we next evaluated whether CD54 is involved in the sensing of SARS-CoV-2-infected cells by pDCs (Fig. 5A). Using an Ab-mediated receptor blockade assay, we found that blocking CD54 on PBMCs reduced the level of IFN-I release in a dose-dependent manner, with approximately 75% inhibition at 5 µg/mL of αCD54 Ab (Fig. 5B). To ensure that this effect of blocking CD54 engagement was specific to pDCs, we performed the experiment with purified pDCs (Fig. S4A). Consistent with what was observed with PBMCs (Fig. 5B), blocking CD54 on pDCs attenuated IFN-I production in a concentration-dependent manner (Fig. S4B). Having confirmed that HeLa-hACE2 cells express a high level of CD54 (Fig. 4A), we asked whether blocking the receptor on infected cells would have a similar effect on IFN-I release. Indeed, when SARS-CoV-2 infected HeLa-hACE2 were pre-incubated with αCD54 Ab before co-culture with PBMCs, IFN-I level was also reduced in a concentration-dependent fashion (Fig. 5C). Of note, these findings were not unique to HeLa-hACE2 cells since the same phenotype was also observed when the blocking experiments were done in the Calu-3 cell system (Fig. 5D and E; Fig. S4C). Furthermore, we excluded the possibility that the αCD54 Ab might have non-specifically affected the TLR7 signaling pathway, through which pDCs sense viral RNA (Fig. S4D).

Conversely, we explored the effect of blocking CD11a on PBMCs and infected cells on pDC sensing (Fig. 5F). To this end, pre-treating PBMCs with αCD11a Ab also meaningfully decreased IFN-I secretion (Fig. 5G). Interestingly, blocking CD11a on both PBMCs and SARS-CoV-2-infected cells affected more drastically immune sensing as shown by a greater reduction in the IFN-I release at all concentrations tested (compare Fig. 5G and H). It should be mentioned that the αCD11a Ab did not negatively affect pDC activation through the TLR7 signaling pathway (Fig. S4E). Taken together, our results reveal that CD54 and CD11a engagement contribute to the recognition and sensing of SARS-CoV-2 infected cells by pDCs, notably when interactions of infected cells with PBMCs promote the induction of CD11a. The data further demonstrate that the potential bidirectional CD54/CD11a cross-talks promote efficient sensing of SARS-CoV-2-infected cells by pDCs.

### Formation of conjugates between pDCs and SARS-CoV-2 infected cells is dependent on receptor engagement with CD54

**Fig 6:**
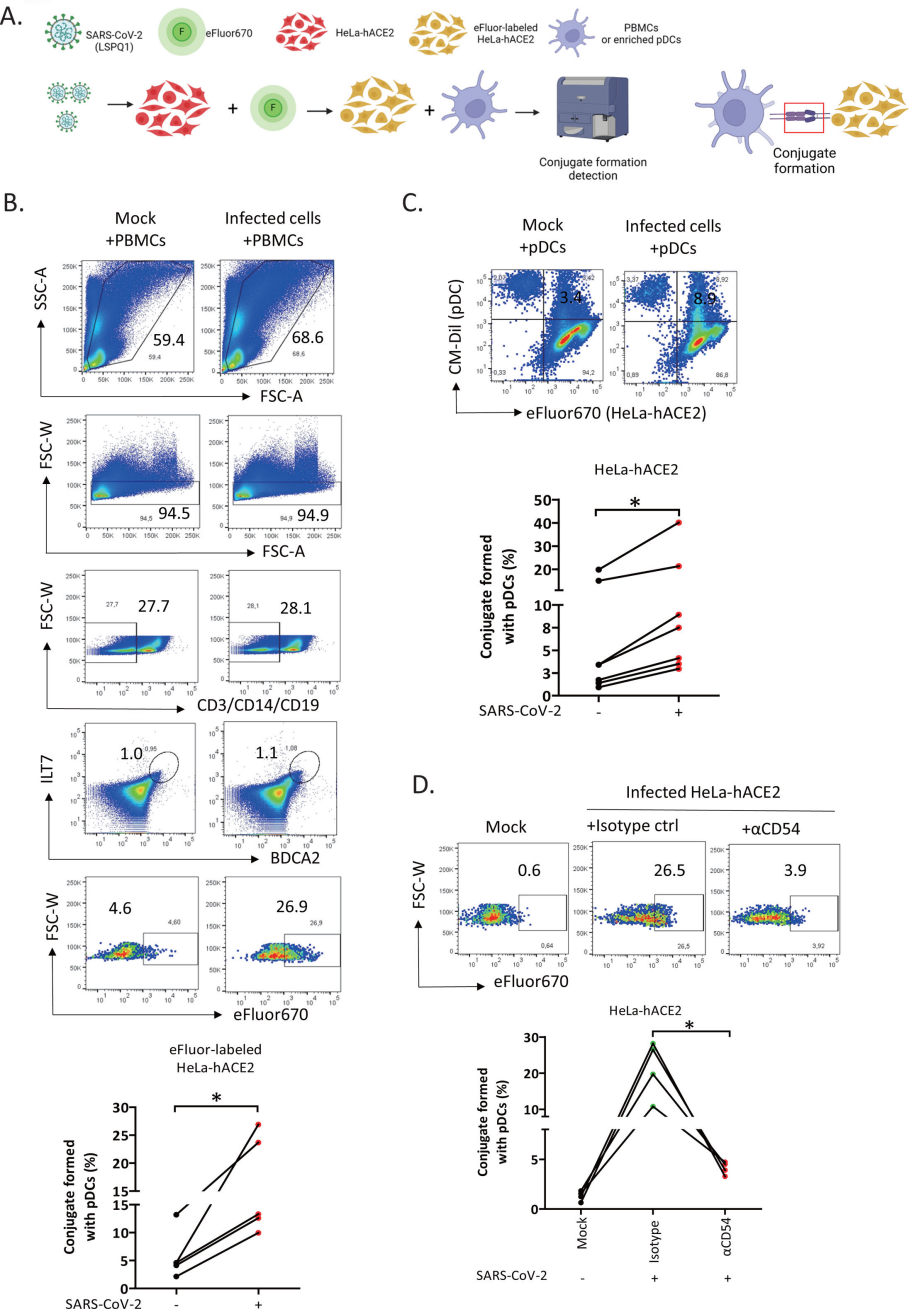
Formation of conjugates between pDCs and SARS-CoV-2-infected cells is dependent on receptor engagement with CD54. (**A**) Schematic representation of the experimental design (created with BioRender.com). Mock- and SARS-CoV-2-infected HeLa-hACE2 were pre-labeled with cell-membrane dye eFluo 670 before co-culturing with PBMCs. Cell conjugates were analyzed by flow cytometry and defined as CD3^-^CD14^-^CD19^-^ILT7^+^BDCA2^+^eFluor670^+^ population. Analysis was done 18–24 h after the co-culture. (**B**) (Upper panel) Dot plots showing the gating strategy to identify cell conjugates between pDCs (gated from PBMCs) and mock- or SARS-CoV-2-infected HeLa-hACE2. Numbers shown in the dot plots are cell percentages. (Lower panel) Frequency of cell conjugates from different experiments done with five donors. (**C**) (Upper panel) Representative dot plots showing the frequency of conjugates formed between enriched pDCs (CM-Dil^+^) and mock- or SARS-CoV-2-infected HeLa-hACE2 (eFluor 670^+^). (Lower panel) Overall data from different experiments done with 7 PBMC donors. (**D**) (Upper panel) Representative dot plots showing the effect of blocking CD54 receptor engagement on conjugate formation between pDCs and infected HeLa-hACE2. Numbers indicated represent the percentage of conjugates. (Lower panel) Frequencies of conjugates between pDCs and eFluor670-labelled mock- and SARS-CoV-2-infected HeLa-hACE2 in the presence of αCD54 Ab or isotype control (5 µg/mL). Analysis was done with four donors. In all relevant panels, each line depicts a donor. Statistical analysis Mann-Whitney *U* test. *P < 0.05. See also Fig. S5 and S6.

Given that pDCs have been shown to form long-lasting contacts, or conjugates, with Dengue virus-infected cells compared with short-lived contacts with uninfected cells (18), we asked whether the same could be observed with SARS-CoV-2-infected cells. For this purpose, mock- or SARS-CoV-2-infected HeLa-hACE2 were pre-labeled with cell membrane dye eFluor 670 prior to being co-cultured with PBMCs or purified pDCs (Fig. S5A; Fig. 6A). In the analysis with PBMCs, pDCs were identified as CD3^-^CD14^-^CD19^-^BDCA2^+^ILT7^+^, and in this context, the mean frequency of conjugate formation between pDCs and infected HeLa-hACE2 was on average 2.9-fold higher than that with mock-infected cells (Fig. 6B, lower panel). Of note, expression levels of the pDC-specific markers BDCA2 and ILT-7 (28, 29) tend to decrease over the 24 h culture period, likely explaining the dimmer expression of BDCA2 and ILT-7 on pDCs at the end of the co-culture (Fig. S5B; Fig. 6B). This could also potentially explain the absence of clearly distinct BDCA2^+^ILT7^+^ pDC populations based upon gating with isotype controls for both αBDCA2 and αILT7 Abs (Fig. S6A). Interestingly, we observed that the use of a fluorescence minus one (FMO) control for only the αILT7 Ab enabled the identification of CD3^-^CD14^-^CD19^-^BDCA2^+^ pDCs. This population formed conjugates more frequently with infected HeLa cells compared with mock-infected cells (Fig. S6B), albeit to a lesser degree as shown in Fig. 6B (a median increase of 1.6-fold compared with 2.9-fold). Variability among donors and between experiments might have contributed to the differences observed. Of note, analysis done using purified pDCs that had been labeled with the cell-tracker dye CM-Dil revealed a statistically significant increase in the double-positive population (eFluor 670^+^ CM-Dil^+^) in infected cells compared with uninfected cells (Fig. 6C), thus validating earlier data obtained with total PBMCs (Fig. 6B).

In light of our earlier results showing that CD54 blockade negatively affected pDC sensing of infected cells, resulting in reduced IFN-I release (Fig. 5), we assessed whether CD54 participates in the formation of conjugates. Indeed, blocking CD54 engagement during the co-culture significantly reduced the frequency of conjugate formation between pDCs and infected cells (Fig. 6D). Together, our data demonstrate that pDCs form significantly more conjugates with SARS-CoV-2-infected cells and that CD54 plays an important role in this process.

### Conjugate formation plays a role in differential pDC-mediated sensing of cells infected with SARS-CoV-2 VOCs

**Fig 7:**
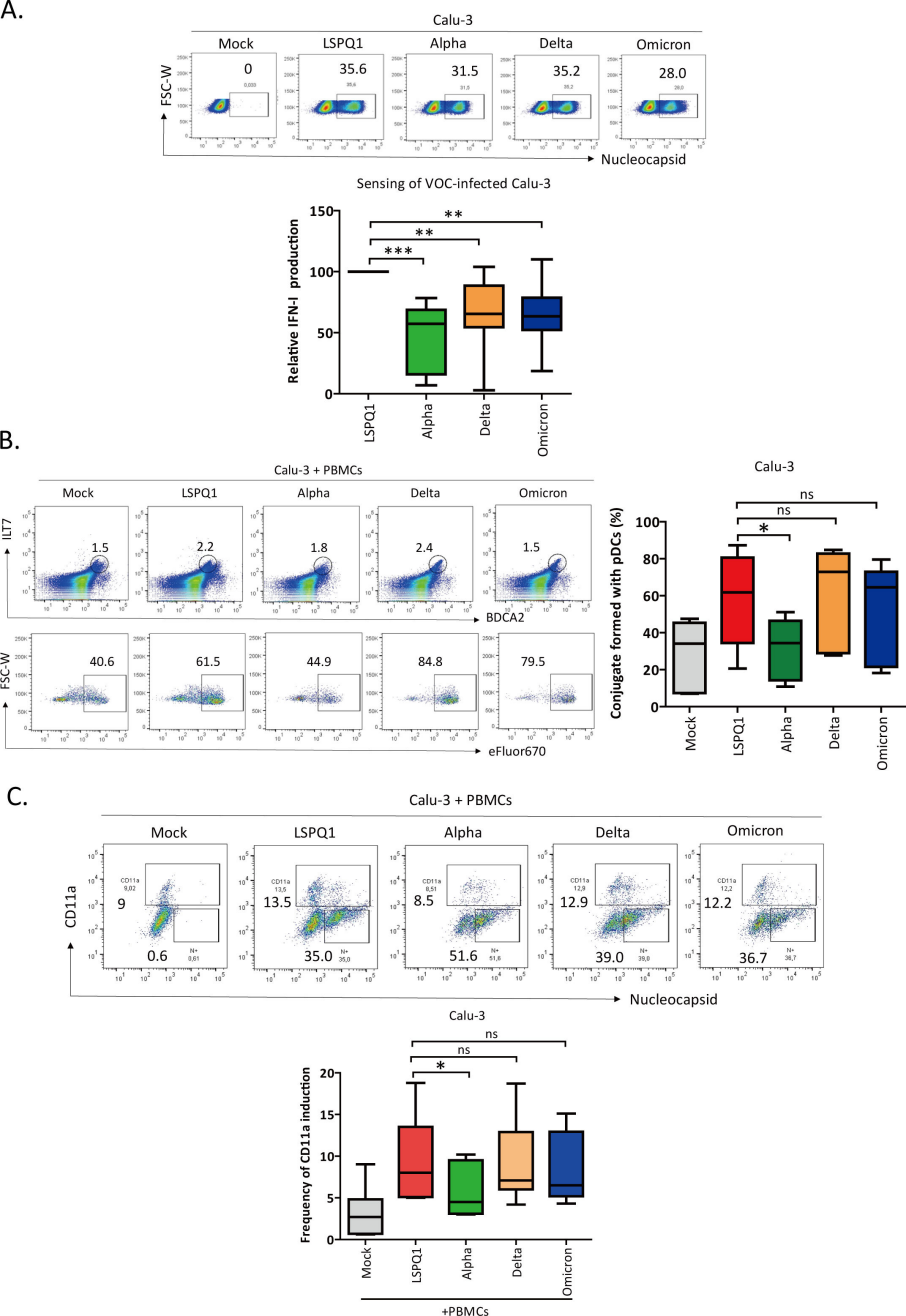
Conjugate formation plays a role in differential pDC-mediated sensing of cells infected with SARS-CoV-2 VOCs. (**A**) (Upper panel) Representative dot plots showing similar SARS-CoV-2 VOC infection rates in Calu-3 at the time of the co-cultures with PBMCs (24 h post-infection). The infection rates were determined by flow cytometry using αN Ab. Numbers indicate percentages of N-positive cells. (Lower panel) IFN-I released from the supernatant of PBMCs co-cultured for 24 h with Calu-3 infected with different VOCs. IFN-I production detected from sensing of LSPQ1-infected Calu-3 was set at 100%. The data were compiled from experiments with 11 PBMC donors. Wilcoxon signed rank test. **P < 0.01; ***P < 0.001 (**B**) (Left panel) Representative dot plots showing conjugate formation between pDCs (gated from PBMCs, defined as CD3^-^CD14^-^CD19^-^ILT7^+^BDCA2^+^) and mock or infected Calu-3 labeled with the eFluor 670 dye. Numbers on the dot plots at the top indicate the frequency of pDCs, while those on the bottom dot plots depict the percentage of conjugates formed between pDCs and eFluor670-labelled Calu-3. (Right panel) Frequency of cell conjugates obtained from experiments with six distinct donors. Wilcoxon signed rank test. *P < 0.05, ns, not significant. (**C**) (Upper panel) Human lung epithelial cells were left uninfected or infected for 24 h with SARS-CoV-2 VOCs and then subjected to a co-culture with PBMCs for another 24 h. Shown is a representative dot plot depicting the percentage of CD11a-positive epithelial cells after the co-cultures. (Lower panel) Compiled data from experiments done with 7 PBMC donors. Wilcoxon signed rank test. *P < 0.05, ns, not significant. In all relevant panels, the data are shown with Whisker plots. The median, minimum and maximum values are as indicated. See also Fig. S3, S5, S7, S8, and S9.

SARS-CoV-2 has produced numerous variants since the first outbreak of COVID-19 in late 2019. These VOCs have evolved and displayed enhanced transmissibility (30) and immune evasion capacity (31). We thus asked whether the increased transmissibility of SARS-CoV-2 VOCs might be related to differences in sensing and IFN-I production by pDCs. To this end, Calu-3 were infected with selected variants known as the Alpha (B.1.1.7), Delta (B.1.617.2), and Omicron (B.1.1.529) along with the Wuhan-similar LSPQ1 strain at different MOIs for 24 h. Infected cultures with similar infection rates were exposed to PBMCs for 24 h, and the supernatant from the co-cultures was measured for IFN-I. To this end, we show that PBMC sensing of infected cells varies among the different VOCs (Fig. 7A; Fig. S7). At similar infection rates, sensing of LSPQ1-infected cells was most efficient and significantly higher compared with the three VOCs tested (Fig. 7A; Fig. S7A). No significant differences in the infection rates among the VOCs were observed over the 24 h co-culture period (Fig. S7B).

To ascertain whether the differences in the IFN-I release among the VOCs might be related to variations in the frequency of conjugate formation, we examined the ability of pDCs to form conjugates with cells infected with these variants. Here, we observed that at comparable infection rates (Fig. 7A; Fig. S7A), conjugate formation between pDCs and Alpha variant-infected Calu-3 was approximately 2-fold less frequent compared with that formed with cells infected with the LSPQ1, Delta, and Omicron variants (Fig. 7B; Fig. S5A). Importantly, the inclusion of isotype controls for both αBDCA2 and αILT7 Abs in flow cytometry analysis showed the same trend of results, although the absolute frequencies of conjugates formed were lower in two out of three donors used in the new experiments (Fig. S8A). Indeed, we still observed impaired conjugate formation between pDCs and Alpha variant-infected Calu-3 (Fig. S8A).

As well, when the experiments were performed with purified pDCs instead of PBMCs, the frequency of pDCs forming cell conjugates with Alpha-variant infected cells was also lower compared with the other variants (Fig. S8B through D). Interestingly, there was about 2-fold less CD11a induction on cells infected with the Alpha variant compared with those infected with the LSPQ1 (Fig. 7C). No significant differences in the level of CD11a induction were observed between the LSPQ1, Delta, or Omicron. Importantly, when flow cytometry analysis was done in the experimental setting where not only PBMCs were excluded from data analysis but also the isotype control for the αCD11 Ab was included, the reduced CD11a expression on Calu-3 cells infected with the Alpha variant was still evident (Fig. S9). Altogether, our results support the notion that SARS-CoV-2 variants that emerged later in the COVID-19 pandemic have developed means to reduce immune sensing of infected cells. The data also highlight the importance of conjugate formation for the release of IFN-I by pDCs, and in this context, the Alpha variant may antagonize this host response by reducing CD11a induction and decreasing conjugate formation. These results further underlie that the potentially bidirectional engagement of CD54 and CD11a facilitates optimal recognition and sensing of SARS-CoV-2 infected cells.

## DISCUSSION

Plasmacytoid DCs are essential mediators of IFN-I responses and as such are critical for the initiation and propagation of successful immunity against viral pathogens. Here, we demonstrate using various cell type models that pDCs are the major producers of IFN-I upon immune sensing of SARS-CoV-2 infection and sensing of infected cells is much more efficient compared with cell-free virions. Mechanistically, we confirm previous data (2) showing that pDC sensing of infected cells requires physical contact and that this process is dependent on the engagement of the intercellular adhesion molecule CD54 and its ligand, CD11a. We uncover that CD11a expression can be induced on human lung epithelial cells, such as Calu-3 during *in-cellulo* interactions with immune cells, and the induction was more efficient compared with uninfected cells. Moreover, our study also reveals that Ab-mediated blocking of CD11a on both PBMCs and infected cells reduces more significantly IFN-I production than blocking PBMCs alone, raising the possibility of bidirectional engagement between CD54 and CD11a. Importantly, our data show for the first time that VOCs have evolved to attenuate sensing of infected cells by pDCs, likely through distinct mechanisms. Indeed, although cells infected with the Delta or Omicron variants did not display measurable defects in CD11a induction and conjugate formation with pDCs, the same can not be said for Alpha variant-infected cells. Their impaired sensing was linked to suboptimal formation of conjugates with pDCs and reduced induction of CD11a on infected cells, thus highlighting one of the mechanisms through which SARS-CoV-2 might limit antiviral response to promote viral replication and spread.

Using a variety of non-polarized and polarized cell systems, as well as differentiated human primary airway epithelial cells, we provide evidence that pDCs are the major source of IFN-I production in response to SARS-CoV-2 infection. Consistent with previous reports (12–14), we find that pDCs can sense cell-free virions. However, we also uncover that sensing of infected cells is significantly more potent as shown by a robustly higher level of IFN-I release (Fig. 1A and Fig. 3). Importantly, we exclude the possibility that the enhancement in IFN release was a direct result of pDCs sensing a greater number of viral particles produced within infected cells (Fig. S1E). The data strongly demonstrate that viruses transmitted through cell-to-cell contact are more potent inducers of IFN-I by pDCs, a finding that was not previously known or investigated. Such differential sensing could be due to variations between cell-free virions and cell-associated viral particles regarding the uptake route, transit, and/or access of the viral genomic RNA in TLR7-associated endosomes. On a relevant note, clathrin-mediated endocytosis has been shown to be involved in pDC sensing of cells infected with other viruses including Dengue, Hepatitis C, or Zika viruses (17, 18). Whether this process is relevant in SARS-CoV-2 infection requires further investigation.

The absence of the main SARS-CoV-2 entry receptor ACE2 on pDCs indicates that viral entry in pDCs is unlikely to be mediated through the binding of the viral protein Spike to the ACE2 receptor. On this note, it remains to be elucidated whether TMEM106B (32) or other receptors that have been proposed to mediate SARS-CoV-2 entry can facilitate the recognition and/or sensing of infected cells by pDCs. Moreover, neuropilin-1 has been shown to potentiate SARS-CoV-2 entry and infectivity, owing to its ability to bind to furin-cleaved Spike protein (33). Interestingly, neuropilin-1 is also known as BDCA-4, a pDC-specific marker, and blocking this receptor has been demonstrated to attenuate pDC sensing of SARS-CoV-2 cell-free virus (14).

PDC recognition of infected cells through physical contact is an important first step toward mounting a response against pathogens (16–18). In agreement with a recent study documenting the necessity of pDCs to interact with SARS-CoV-2-infected cells (2), our data demonstrate that separating infected cells from pDCs reduces IFN-I release by 90%–95% (Fig. 2).

Growing lines of evidence have shown that not only the respiratory epithelium can be infected by SARS-CoV-2 (34) but also the intestinal epithelium is targeted by the virus (21). These epithelia are present in a polarized/differentiated fashion *in vivo* with apical and basolateral domains; immune cells such as pDCs are found on the basolateral side. Using diverse polarized cell models and differentiated primary epithelial cells from the intestine and lung, we recapitulated our earlier findings in unpolarizable HeLa-hACE2 cells (Fig. 2). Indeed, we discovered that direct contact between pDCs and infected cells is critical since cells polarized/differentiated on 0.4 µm transwell pore size were poorly sensed compared with those polarized on a 3.0 µm membrane (Fig. 3; Fig. S2). Like SARS-CoV-1 (22), we show that SARS-CoV-2 releases infectious viral particles mostly from the apical side (Fig. 3), and we further document that the presence of PBMCs does not alter the polarization of infectious virion secretion (Fig. S2A). Importantly, detectable IFN-I was only found on the basolateral side where pDCs were in direct contact with infected cells, a finding that has not been previously reported. There was no detectable IFN-I produced by polarized infected cells in our experimental systems, emphasizing once again the important contribution of pDC sensing infected cells to IFN-I production following SARS-CoV-2 infection.

Our data show that a principal requirement for the immunostimulatory signal on the basolateral side to trigger the IFN-I release is the direct contact between pDCs and infected cells. Although the amounts of virus released from the basolateral are exceedingly low compared with those from the apical side, they are still detectable. This indicates that infected cells can display viruses from the basolateral surface, and in the context of conjugates with pDCs, the viruses could be transmitted. The fact that virus transmitted via cell-to-cell contact induces more potently IFN-I production compared with cell-free virions suggests that these low amounts of virus transmitted through cell-to-cell contact can provide an immunomodulatory signal to trigger pDC response. Overall, our data suggest that it is unlikely for cell-free virus/viral RNA or soluble factors that may be released from the basolateral side to contribute to the sensing of infected cells by pDCs since the supernatant from polarized infected cells does not trigger a pDC response (Fig. 3).

Multiple studies have indicated that pDCs express abundantly CD11a and its ligand CD54, whereas virus-infected cells express only CD54 (2, 18), suggesting that physical contact of pDCs and infected cells is mediated by a directional engagement of CD54 on infected cells to CD11a on pDCs. Our data regarding surface expression of CD54 and CD11a agree with these studies. However, we also establish that CD11a expression is inducible on SARS-CoV-2 susceptible cells and that the possibility of the CD11a signal coming from random conjugates between PBMCs and Calu-3 was excluded or at least, minimized. In addition, we find that CD11a induction requires direct contact between infected cells and PBMCs (Fig. 4). Removing PBMCs during analysis did not significantly change the level of CD11a^+^ cells, indicating that CD11a induction was mainly on CD3^-^CD14^-^CD19^-^HLA-DR^-^CD11c^-^BDCA2^-^ILT7^-^ cells. Our study reveals that although pDCs alone are unable to stimulate CD11a expression and monocytes by themselves upregulate only modestly the level, depleting either cell subtype from PBMCs significantly reduced the extent of CD11a induction (Fig. 4E; Fig. S3C). Consistent with previous report (2), we find that CD54 and CD11a interactions are important in the sensing of infected cells. However, our study also documents that pre-blocking CD54 on either pDCs/PBMCs or infected cells reduces IFN-I release in a dose-dependent manner (Fig. 5). As well, we uncover that blocking CD11a on both PBMCs and infected cells leads to a more pronounced decrease in IFN-I production compared with blocking PBMCs alone (Fig. 5). Thus, our findings collectively raise the possibility of bidirectional cross-talks between CD54 and CD11a whereby a pDC and an infected cell initially make contact through an interaction between CD11a on the pDC and CD54 on the infected cell. Following formation of this conjugate, a secondary contact with monocyte(s) triggers the induction of CD11a on infected cells, thus further strengthening the cell synapse and promoting optimal sensing and IFN-I production by pDCs. This said, further experimentation with SARS-CoV-2 susceptible cells depleted of these cell adhesion molecules would be needed to strengthen the argument of bidirectional interactions. As well, it remains to be established whether there is a colocalization of cells expressing viral proteins, CD11a and CD54, and whether the process involves cell conjugates made between infected cells, pDCs, and monocytes.

In the context of the interferogenic synapse, our study illustrates that pDCs can form conjugates with both uninfected and infected cells, although such conjugate formation was significantly more frequent on infected cells (Fig. 6; Fig. 7). The data indicate that CD54 and CD11a are directly involved in this process since Ab-mediated blocking of the CD54 receptor or decreased level of CD11a drastically reduce the formation of conjugates (Fig. 6; Fig. 7).

Recent studies have shown that SARS-CoV-2 variants evolved from the original ancestral strain and emerged later in the COVID-19 pandemic, have developed different means to delay and attenuate the IFN response in infected cells (31, 35). By modulating the expression and function of viral gene products, notably ORF6, ORF9b, and N, these variants were found to have acquired an enhanced capacity to antagonize the IFN response in target cells (31, 35). Our findings here-in reveal an additional strategy that VOCs could limit the level of IFN-I production and minimize the establishment of an antiviral state in target cells. However, the underlying mechanism(s) behind the defect appear to be different among the VOCs. In the case of cells infected with the Delta or Omicron variants, although the extent of conjugate formation and the level of CD11a induction was comparable with LSPQ1-infected cells, IFN-I production was still significantly less. In the case of the Alpha variant, blunted CD11a induction on infected cells decreases pDC sensing leading to inefficient IFN-I release, a condition that will lead to suboptimal establishment of an antiviral state within target cells. This in turn contributes to enhanced transmissibility as reported with this variant compared with the original SARS-COV-2 (36). This being said, there might be other factors that could potentially influence pDC responses. Indeed, differences in viability or metabolic activity of infected cells among the VOCs could also indirectly modulate pDC sensing. In our experimental conditions, we do not observe marked differences in cell viability between Wuhan-like LSPQ1 and the VOCs at any time point during the co-cultures. Given that SARS-CoV-2 has been shown to affect metabolic activity of infected cells (37), it remains to be determined whether the virus has evolved to impact this process differentially among the VOCs. As well, Venet and colleagues (2) have shown that upon contact with cells infected with a SARS-CoV-2 infectious molecular clone, pDCs induce a higher level of IRF7/IFN-I-regulated cytokines than NF-κB regulated TNFα. Thus, it remains to be established whether these pathways are triggered differently by VOCs to ultimately modulate the IFN response by pDCs.

Overall, the present study characterizes some of the early steps involved in pDC-mediated response against SARS-CoV-2 infection. We provide a mechanistic insight into the molecular mechanism underlying sensing of infected cells by pDCs and show that SARS-CoV-2 has evolved to antagonize this process to limit IFN-I antiviral responses. These findings advance our understanding of the pathogenesis of SARS-CoV-2.

## MATERIALS AND METHODS

### Cell lines and primary cell cultures

Human lung epithelial Calu-3 cells (derived from a male) and human intestinal epithelial Caco-2/15 cells (derived from a male), kindly provided by Dr. Emile Levy (Research Center of the Centre Hospitalier Universitaire Sainte Justine [CHUSJ], Montreal, Canada), were maintained in Eagle’s Minimum Essential Medium (EMEM) supplemented with 10% FBS. HEK-BlueIFN-α/β human IFN-I reporter cell line was cultured in Dulbecco’s Modified Eagle’s Medium (DMEM) supplemented with 10% FBS. Caco-2/15 and Calu-3 were polarized on 0.4 µm or 3.0 µm pore size transwell membrane inserts (Sterlitech). Cell polarization was determined via evaluation of the transepithelial electrical resistance (TEER, Millipore Sigma). Briefly, the TEER value was calculated by subtracting the electrical resistance of the filter insert in the absence of epithelial cells from the value measured in the presence of confluent cell monolayers, and then by multiplying the result with the surface area of the insert. As a measure of TEER, the resulting unit is given in Ω · cm^2^. TEER values of well-differentiated Calu-3 and Caco-2/15 were >1,000 Ω · cm^2^ and in the range of 380–450 Ω · cm^2^, respectively (38, 39). HeLa cell line stably expressing the full-length human ACE2 receptor (HeLa-hACE2) was generated by transducing HeLa cells with lentiviral vector particles expressing the human ACE2 and blasticidin-resistance genes (the lentiviral vector pWPI-IRES-Bla-Ak-ACE2-TMPRSS2 was a kind gift from Dr. Sonja Best, Addgene plasmid #154983). The cell line was maintained in DMEM supplemented with 10% FBS and 50 µg/mL of blasticidin (InvivoGen). Vero E6 cell line, kindly provided by Dr. Nabil Seidah, was maintained in DMEM supplemented with 10% FBS. Vero E6 stably expressing the full-length human TMPRSS2 receptor (Vero-TMPRSS2) was generated by transducing Vero E6 cells with lentiviral vector particles expressing the human TMPRSS2 and neomycin-resistance genes (the lentiviral vector RRL.sin.cPPT.SFFV/TMPRSS2 (variant 1).IRES-neo.WPRE (MT130) was a generous gift from Dr. Caroline Goujon (40), Addgene plasmid #145843). Vero-TMPRSS2 cells were cultivated in DMEM containing 10% FBS and 1 mg/mL G418 (InvivoGen).

Peripheral blood samples were obtained from healthy adult donors who gave written informed consent following the Declaration of Helsinki and under a research protocol approved by the research ethics review board of the IRCM. Peripheral blood mononuclear cells (PBMCs) were isolated by Ficoll-Paque centrifugation (GE Healthcare) and cultured in RPMI-1640 media supplemented with 10% FBS. In certain experiments, pDCs were depleted from total PBMCs by flow cytometry cell sorting following multiparametric staining with FITC-conjugated Abs αCD3, αCD14, and αCD19 as well as BV421-conjugated αBDCA2 and PE-conjugated αILT7 Abs. BV421-conjugated mouse IgG2a and PE-conjugated mouse IgG1 were used as isotype controls for the αBDCA2 and αILT7 Abs, respectively. The Abs and isotype controls were from BioLegend. PDCs were enriched by positive selection using the Miltenyi Biotec Human Diamond Plasmacytoid Dendritic Cell Isolation Kit II. Enriched pDCs, with a typical purity of greater than 95%, were also cultured in RPMI-1640 media supplemented with 10% FBS.

Primary human bronchial epithelial cells (HBECs) were obtained from the Respiratory Cell and Tissue Biobank of the Centre de Recherche of the Centre Hospitalier de l’Université de Montréal (CRCHUM/CHUM) affiliated with the Quebec Respiratory Health Research Network with informed written consent before enrolment and approval of the research study by the CR-CHUM Institutional Review Board (CER 08.063). HBECs were isolated from bronchial biopsies collected from three healthy subjects (B-GD-172: male; B-GD-180: female; B-GD-175: female) and cultured as previously described (41) on transwell permeant inserts (Costar) coated with collagen IV (Sigma) and PureCol (Advanced BioMatrix), in a mixture (50:50) of PneumaCult Ex and CnT-17 airway epithelial proliferation medium (CellNTec Advanced Cell System) for approximately 2 days, and then in CnT-17 airway epithelial proliferation medium to confluence. Cell cultures were then placed at the air-liquid interface with the basolateral side bathed with a mixture (1:1) of DMEM and Bronchial Epithelial Cell Growth Medium (BEGM), supplemented with bronchial epithelial SingleQuots^TM^ kit (Lonza) containing hydrocortisone (0.5 mg/mL), insulin (5 mg/mL), transferrin (10 mg/mL), epinephrine (0.5 mg/mL), triiodothyronine (6.5 mg/mL), amphotericin-B (50 mg/mL), retinoic acid (0.1 ng/mL), human recombinant epidermal growth factor (0.5 ng/mL), penicillin/streptomycin (50 U/mL) and BSA (1.5 µg/mL) for 5 weeks until full differentiation.

### Preparation of SARS-CoV-2 stocks and cell infections

The prototypic SARS-CoV-2 variant used in this study is LSPQ1 (B1 lineage, GISAID: EPI_ISL_535728). This isolate is similar to the Wuhan strain (42). LSPQ1 was isolated from a 51-year-old female in Quebec by the Laboratoire de Santé Publique du Québec (LSPQ). The Alpha variant (B.1.1.7, GISAID: EPI_ISL_683466) was obtained from BEI Resources. The Delta variant (B.1.617.2, ID: L00414346/COV2021-0248 3384976) was obtained from the National Microbiology Laboratory (Winnipeg, Canada). LSPQ1, Alpha (B.1.1.7), and Delta (B.1.617.2) variants were amplified and titrated in Vero E6 cells using a previously described plaque assay (43). The Omicron (B.1.529, ID: L00423388) variant, obtained from LSPQ, was amplified once in Calu-3 cells and titrated in Vero-TMPRSS2 by plaque assay.

PBMCs were exposed to SARS-CoV-2 cell-free virus (MOI 0.1, for 24 h). HeLa-hACE2 and unpolarized Calu-3 cells were infected with LSPQ1 (MOI 0.1) for 24 h, unless otherwise indicated. Polarized Caco-2/15 and Calu-3 cells were infected with LSPQ1 (MOI 0.1, for 48 h) from the apical domain. Differentiated HBECs were infected with LSPQ1 (MOI 5, for 72 h) from the apical side. Calu-3 cells were infected with LSPQ1, or VOCs Alpha, Delta, and Omicron at different MOIs ranging from 0.001 to 0.5 for 24 h to obtain a condition whereby the level of nucleocapsid-expressing cells, as determined by flow cytometry, was similar between the four strains of SARS-CoV-2.

### Plaque assay

Plaque assay was conducted as previously described (43) with modifications. Briefly, Vero E6 or Vero-TMPRSS2 were seeded in duplicates in a 12-well plate (180,000 cells/well) 2 days before the experiment. Cells were infected with five to six ten-fold serial dilutions of viruses for 1 h at 37°C. The plates were manually rocked every 15 min during the 1 h period. Subsequently, cells were overlayed with 2 mL of a solid media containing 50% DMEM and 50% low melting point agarose (1.2%) (Thermo Fischer Scientific). The plates were left undisturbed for 60 to 65 h at 37°C. Cells were fixed with 4% paraformaldehyde (Sigma-Aldrich) and stained with 0.25% crystal violet (Millipore Sigma, prepared in 30% methanol) following removal of the solid agarose media. Virus titers, expressed as plaque forming units per milliliter (PFU/mL), were determined as follows: (number of plaques x dilution factor of the virus) x 1,000 /volume of virus dilution used for infection (in microliters).

### Assessing the efficiency of SARS-CoV-2 infection by flow cytometry

Infection rates of SARS-CoV-2 in susceptible cells was assessed by flow cytometry using a mouse αN Ab (Sino Biological). Mouse IgG1 was used as an isotype control (BioLegend). Briefly, mock- or SARS-CoV-2-infected cells were stained with Zombie Aqua Live/Dead dye (1/500, BioLegend), followed by permeabilization with BD Cytofix/Cytoperm solution (BD Bioscience) as per manufacturer’s recommendations. Cells were then stained with the mouse αN primary Ab (1/200, Sino Biologicals) or the isotype control mouse IgG1 (1/200, BioLegend) for 30 min at room temperature, followed by staining with a secondary Ab for 30 min at room temperature. Flow cytometry data were acquired on a BD LSR Fortessa flow cytometer (BD Bioscience) and analyzed by Flowjo software (Versions 9.9.3 and 10.1). Infected cells were identified as ZombieAqua^-^ *N*^+^.

### Assessing for cell-surface proteins by flow cytometry

For the detection of CD54 (ICAM-1) or CD11a (α_L_ integrin), cells were incubated with 5 μg/mL PE-conjugated αCD54 Ab (clone M17/4, BioLegend) or PE-conjugated αCD11a Ab (clone HA58, BioLegend), respectively. Parallel staining with relevant PE-conjugated mouse IgG1 (FC) (BioLegend) isotype controls was done to confirm specificity of the staining. In all cases, cells were stained for 30 min at 4°C. For the detection of the hACE2 receptor, cells were stained with 4 µg/mL of goat α-human ACE2 Ab (R&D system) or with the isotype control (goat IgG, R&D system) for 30 min at 4°C, followed by incubation with a secondary Ab. Samples were acquired as mentioned above.

### Co-culture experiments to assess for IFN-I release

Unless otherwise stated, cells were infected for 24 h and then co-cultured with PBMCs for 18–24 h. In brief, 1 × 10^4^ of HeLa-hACE2 or 2 × 10^4^ of Calu-3 were infected with LSPQ1 in a 96-well plate. Freshly isolated PBMCs or pDC-depleted PBMCs were then added to mock- or SARS-CoV-2-infected cells at a ratio of 10:1 (targets:donors) in a final volume of 200 µL. For sensing experiments in the presence of transwell inserts, HeLa-hACE2 was seeded in the lower chamber of a 24-well plate and infected with LSPQ1. PBMCs were added to the upper chamber of the transwell insert. For experiments involving Calu-3, Caco-2/15, and HBECs, cells were polarized/differentiated in a 24-well plate on transwell membrane inserts. Following infection, PBMCs were subsequently seeded in the lower chamber in a final volume of 600 µL. IFN-I in the supernatant was quantified as described below.

### Co-culture experiments to assess for cell-surface CD11a expression

Calu-3 cells (3 × 10^5^ cells seeded in a 12-well plate) were infected with LSPQ1, Alpha, Delta and Omicron variants for 24 h. Freshly isolated PBMCs were co-cultured with uninfected (mock) or infected cells at a ratio of 10:1 in a final volume of 1 mL for about 18–24 h. After the co-culture, PBMCs were removed, Calu-3 was washed with PBS, detached with PBS/EDTA and assessed for CD11a and SARS-CoV-2 nucleocapsid by flow cytometry, as described above.

To exclude the possibility of PBMC contamination after co-culture, Calu-3 was pre-blocked with 10% healthy human sera diluted in PBS for 30 min at 4°C, followed by staining with a cocktail of Abs: αCD3 and αCD14 (both conjugated with APC-Cy7) (1/200, BioLegend), αCD19 conjugated with PE/Dazzle (1/200, BioLegend), αHLA-DR conjugated with BV605 (1/200, BioLegend), αCD11c conjugated with BV510 (1/200, BioLegend), αBDCA2 conjugated with BV421 (1/200, BioLegend) and αILT7 conjugated with Alexa Fluor 647 (1/200, BioLegend) as well as αCD11a conjugated with PE (1/200, BioLegend). PBMCs were gated out during analysis for CD11a induction on Calu-3.

In experiments aimed to elucidate how CD11a expression was augmented on Calu-3 during co-cultures, purified cell subsets or PBMCs depleted of a given cell type were co-cultured with uninfected or infected (for 24 h) Calu-3 for up to 24 h. In brief, pDCs (human diamond plasmacytoid dendritic cell isolation kit II, Miltenyi Biotec), CD14^+^ monocytes (human CD14^+^ MicroBeads UltraPure, Miltenyi Biotec), and CD19^+^ B cells (Human CD19^+^ MicroBeads, Miltenyi Biotec) were enriched from PBMCs by positive selection. CD4^+^ T cells (Human CD4^+^ T Cell isolation kit, Miltenyi Biotec) were purified by negative selection according to manufacturer’s instructions. In complementary analyses, PBMCs were depleted of monocytes or B cells using magnetic microbeads CD14 and CD19, respectively, or of BDCA2^+^ILT7^+^ pDCs by flow cytometry cell sorting. The exact number of immune cells (purified subtypes or fractionated PBMCs) added to a co-culture was determined based on the sorting frequency as follows: number of specific purified subset = total PBMCs x specific subset frequency. Number of subtype-depleted fractionated PBMCs = total PBMCs x (100% – depleted cell type frequency).

### Quantification of IFN-I

Detection of bioactive IFN-I (IFN-α/β) was conducted using the HEK-Blue IFN-α/ β reporter cell line (InvivoGen), as previously described (44).

### Blocking assays

For blocking experiments with αCD54 Ab, target cells (PBMCs or enriched pDCs) or donor cells (infected HeLa-hACE2 or Calu-3) were pre-treated with αCD54 Ab (0, 0.625, 1.25, 2.5, 5 µg/mL, Bio-Rad) for 30 min at room temperature prior to the 18–24 h co-culture. For blocking experiments with αCD11a Ab, PBMCs were pretreated with αCD11a or αCD11a Ab (0, 2.5, 5, 10 µg/mL, Invitrogen) was added to the co-culture containing PBMCs and infected Calu-3. The resulting supernatant was collected at 18–24 h post-co-culture and assayed for IFN-I level as described above.

### Conjugate assay

Mock- or SARS-CoV-2 VOC-infected HeLa-hACE2 or Calu-3 (donor cells) were labeled with cell membrane dye eFluor670 (1/1000, Invitrogen) for 10 min at 37°C and co-cultured for 18–24 h with PBMCs or CM-Dil (1/1000, Invitrogen)-prelabeled pDCs (target cells) (ratio 1:10, donors:targets, total volume 200 µL). To assess the involvement of CD54 in the formation of conjugates between pDCs and infected cells, αCD54 Ab (5 µg/mL, Bio-Rad) or isotype Ab control (5 µg/mL, BioLegend) was added to the HeLa-hACE2 cell-PBMC co-culture. Cell conjugates, namely HeLa-hACE2 -PBMC, Calu-3 -PBMC, and HeLa-hACE2 -pDC were fixed with 4% PFA, and stained with pDC markers (αCD3, αCD14, αCD19, αBDCA2, and αILT7) at 4°C for 30 min. Samples were acquired on a Fortessa flow cytometer. Data were analyzed using Flowjo and conjugates were identified as CD3^-^CD14^-^CD19^-^BDCA2^+^ILT7^+^ eFluor 670^+^ or CM-Dil^+^ eFluor 670^+^.

### Quantification and statistical analysis

All statistical analysis was conducted by GraphPad Prism5. Data were presented as mean values ± standard error of the mean (SEM), unless otherwise stated. Figure legends report the number of independent experiments or donors, as appropriate. Unless indicated differently, two-tailed Mann-Whitney *U* test was used to calculate *P* values for continuous, non-parametric variables. To non-parametrically compare two populations, Wilcoxon signed-rank test was used. The *P* value of <0.05 was considered statistically significant and *P* value of > 0.05 was depicted as not significant (ns). The *P* value with one star (*) attached is less than 0.05; two stars (**) are less than 0.01, and three stars (***) are less than 0.001.

## Data Availability

All data reported in this article are available without restriction upon written request to the corresponding author.

## SUPPLEMENTAL MATERIAL

Supplemental figures jvi.01235-24-s0001.pdf Figures S1 to S9.

ASM does not own the copyrights to Supplemental Material that may be linked to, or accessed through, an article. The authors have granted ASM a non-exclusive, world-wide license to publish the Supplemental Material files. Please contact the corresponding author directly for reuse.
