# Supplementary material for: Sensing of SARS-CoV-2-infected cells by plasmacytoid dendritic cells is modulated via an interplay between CD54/ICAM-1 and CD11a/LFA-1 αL integrin
Source: J Virol. 2025 Jan 13;99(2):e01235-24. doi: 10.1128/jvi.01235-24 (PMC11852802; doi:10.1128/jvi.01235-24)
Supplement: Supplemental figures — Figures S1 to S9. [file jvi.01235-24-s0001.pdf]

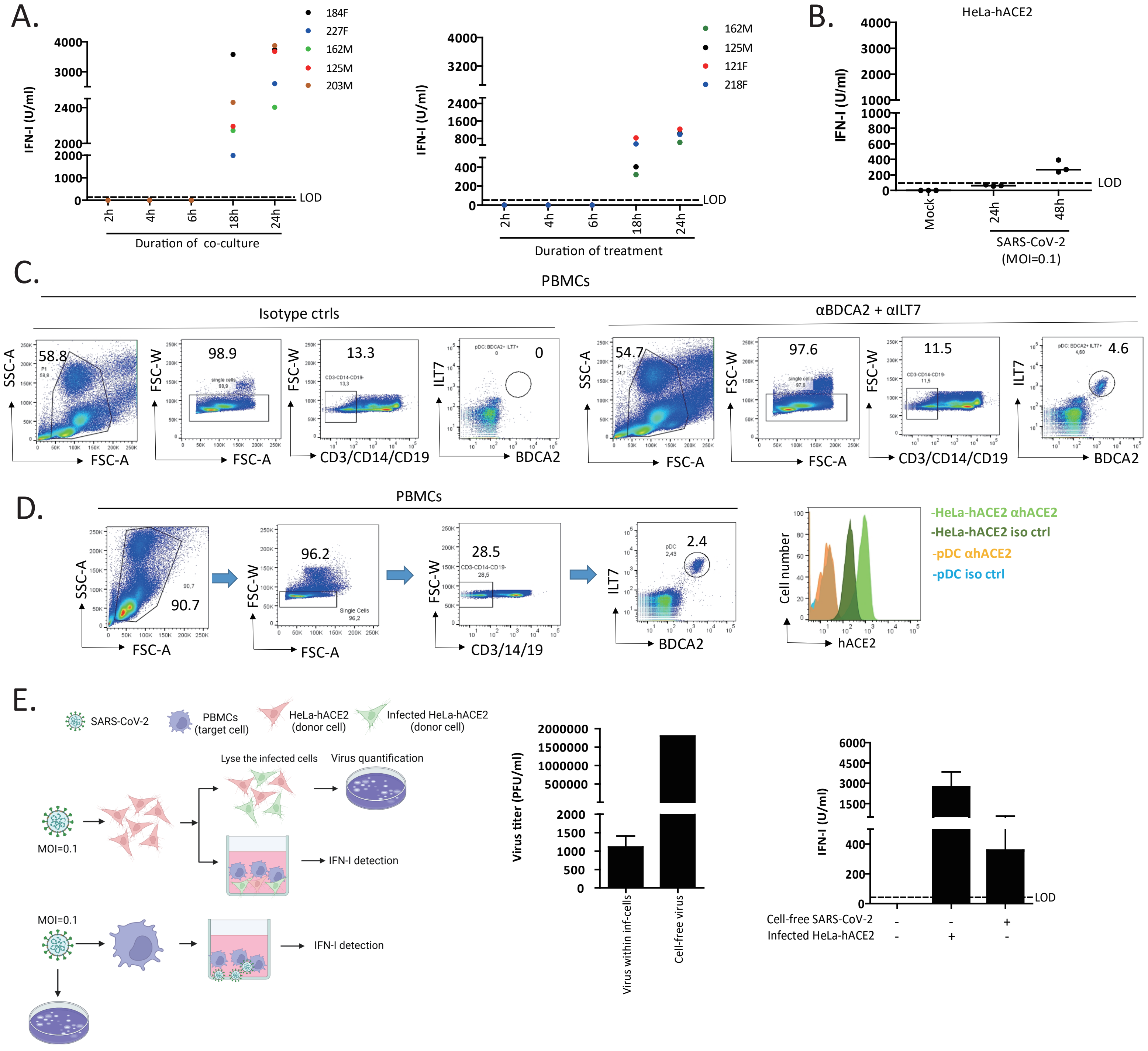

Figure S1. pDC-mediated sensing of SARS-CoV-2-infected cells is more efficient compared to cell-free virions, related to Figures 1 and 2. A. Quantification of IFN-I in the supernatant of PBMCs co-cultured with SARS-CoV-2 HeLa-hACE2 (Left panel, n=5) or treated with cell-free virus (Right panel, n=4) for up to 24 h. Each color dot represents a donor. F, female; M, male. The dotted line indicates the limit of detection (LOD) of the assay (60 U/mL). **B.** Quantification of IFN-I in the supernatant of infected HeLa-hACE2 (MOI 0.1) at 24 h and 48 h in the absence of PBMCs. Errors bars represent the median; each dot represents an independent experiment; n=3. **C.** Shown is the gating strategy to select BDCA2^{+}ILT7^{+} pDCs from PBMCs. BV421-conjugated mouse IgG2a and PE-conjugated mouse IgG1 were used as isotype controls (ctrls) (Left panel) for anti-BDCA2 (αBDCA2) and anti-ILT7 (αILT7) Abs (Right panel), respectively. Shown in the final gate is the frequency of BDCA2^{+}ILT7^{+} pDCs in a true stained sample (4.6%) compared to that stained with the isotype controls (0%). **D.** (Left panel) Representative dot plots showing the gating strategy to gate on pDCs from PBMCs. pDCs are defined as CD3^{-}CD14^{-}CD19^{-}ILT7^{+}BDCA2^{+}. (Right panel) Representative histogram showing human ACE2 receptor (hACE2) expression on pDCs and HeLa-hACE2 (positive control). Cells were stained with anti-hACE2 Ab (αhACE2) or an isotype control (iso ctrl); n=3. **E.** (Left panel) Schematic representation of the experimental design to compare the quantity of infectious virus within infected (inf) HeLa-hACE2 and the viral inoculum used to treat PBMCs. Image was created with BioRender.com. (Middle panel) Quantification by plaque assay of infectious virus within infected HeLa-hACE2 and in cell-free virus (MOI 0.1) that was used to treat PBMCs. Briefly, at 24 h post-infection, LSPQ1-infected HeLa-hACE2 (MOI 0.1) were washed with PBS three times, and 1 mL of serum-free medium was added to the cells, followed by three cycles of freeze-thaw at -80ºC. Then, virus-containing medium was clarified by centrifugation at 3000 x *g* for 10 min. Mean ± SEM; n=3.(Right panel) Quantification of IFN-I in supernatant of PBMCs co-cultured with SARS-CoV-2-infected HeLa-hACE2 (MOI 0.1) or exposed to cell-free virus (MOI 0.1). Mean ± SEM; n=3. The dotted line indicates the LOD.

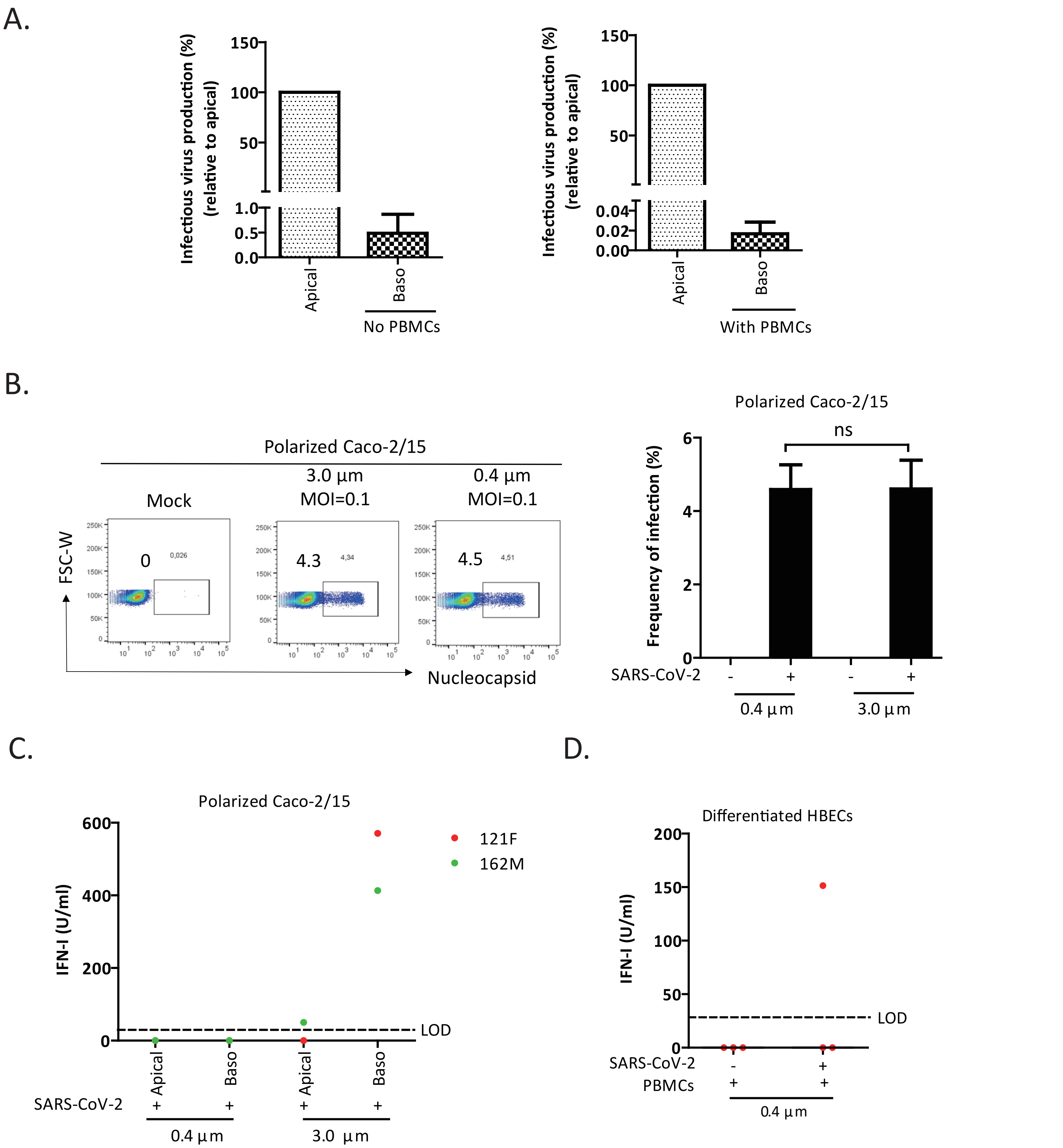

Figure S2. pDCs can sense SARS-CoV-2-infected polarized cell lines and differentiated primary human airway epithelial cells from the basolateral domain, related to Figure 3. A. Directional release of infected polarized Caco-2 (MOI 0.1) in the absence or presence of PBMCs on the basolateral side. The supernatant from the apical and basolateral sides was collected 24 h after co-culturing with PBMCs (72 h infection for Caco-2). Mean ± SEM; n=3. **B.** Infection rate of Caco-2/15 at 48 h (LSPQ1, MOI 0.1) as determined by flow cytometry using anti-N Ab. Cells were polarized on 0.4 µm and 3.0 µm trans-well inserts and infected on the apical side. A representative example of the infection is on the left and compiled data from 3 experiments. Mean ± SEM. Mann-Whitney U test; ns, not significant. **C.** Comparison of the IFN-I release from the supernatant of PBMCs co-cultured with infected Caco-2/15 that were polarized on 0.4 µm and 3.0 µm trans-well inserts before infection. PBMCs were added to the lower chamber facing the basolateral (Baso) domain. Mean ± SEM; each dot is a donor; n=2. **D.** Absolute values of IFN-I released in the supernatant of PBMCs co-cultured with infected HBECs that were differentiated on a 0.4 µm trans-well at the time of infection. PBMCs were added to the lower chamber facing the basolateral domain. Mean ± SEM; n=3. Each dot is a different HBEC.

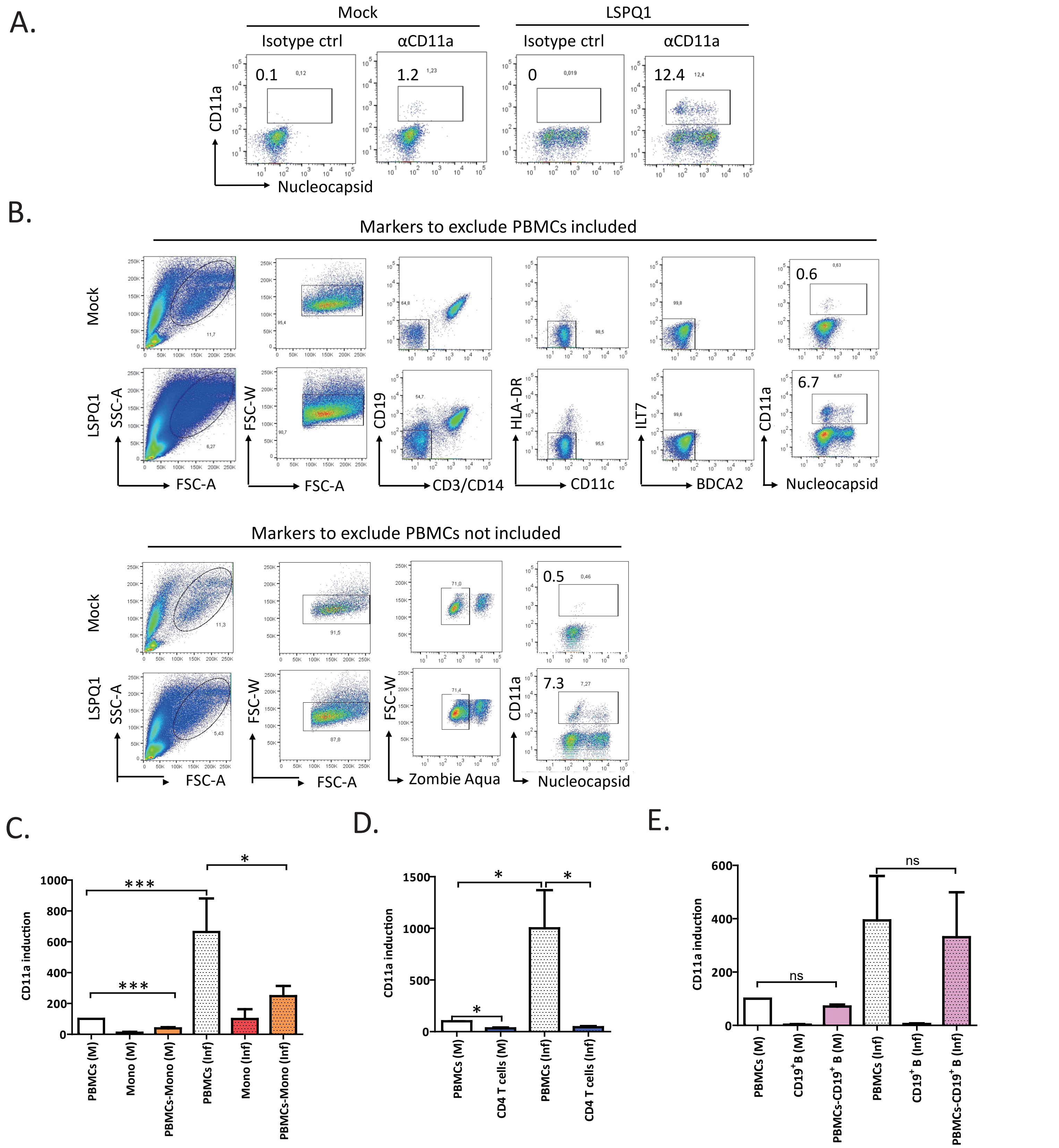

Figure S3. Validating CD11a induction data on Calu-3 with controls. Relative contribution of PBMC subsets to CD11a induction on lung epithelial cells, related to Figures 4 and 7. A. Shown is an example of CD11a induction on uninfected (Mock) and infected Calu-3 (LSPQ1) after co-culture with PBMCs for 24 h. Isotype control (ctrl) for αCD11a: mouse IgG1. **B.** To confirm that CD11a induction on Calu-3 was authentic and not due to CD11a signal coming from PBMCs, flow analysis was done in the presence of Abs specific for different cell subsets in PBMCs (αCD3, αCD14, αCD19, αCD11c, αHLA-DR, αBDCA2, αILT7) (top two panels). The final gate shows percentage of CD11a$^+$ cells defined as CD3$^-$CD14$^-$CD19$^-$CD11c$^-$HLA-DR$^-$BDCA2$^-$ILT7$^-$ CD11a$^+$. The bottom two panel depicts the analysis with the same PBMC donor (158M) where flow staining was performed in the absence of the aforementioned Abs specific for PBMCs. Overall, the data validate the gating strategy and authenticity of CD11a induction on Calu-3. **C-E.** CD11a on Calu-3 after a co-culture with total PBMCs or different cell subsets as indicated. Monocytes (mono) (n=4), monocyte-depleted PBMCs (n=8), CD4$^+$ T cells (n=4), CD19$^+$ B cells (n=3), CD19$^+$ B-depleted PBMCs (n=3). In Panels C, D, and E: CD11a induction was expressed as a percentage of that expressed on mock (M)-infected cells, set at 100 for a given donor. The analysis was done following a 24 h co-culture with total or fractionated PBMCs, meaning that Calu-3 cells were analyzed at 48 h post mock or true infection. Inf, infected. Mean ± SEM; Mann-Whitney *U* test; *$p < 0.05$, ***$p < 0.001$; ns, not significant.

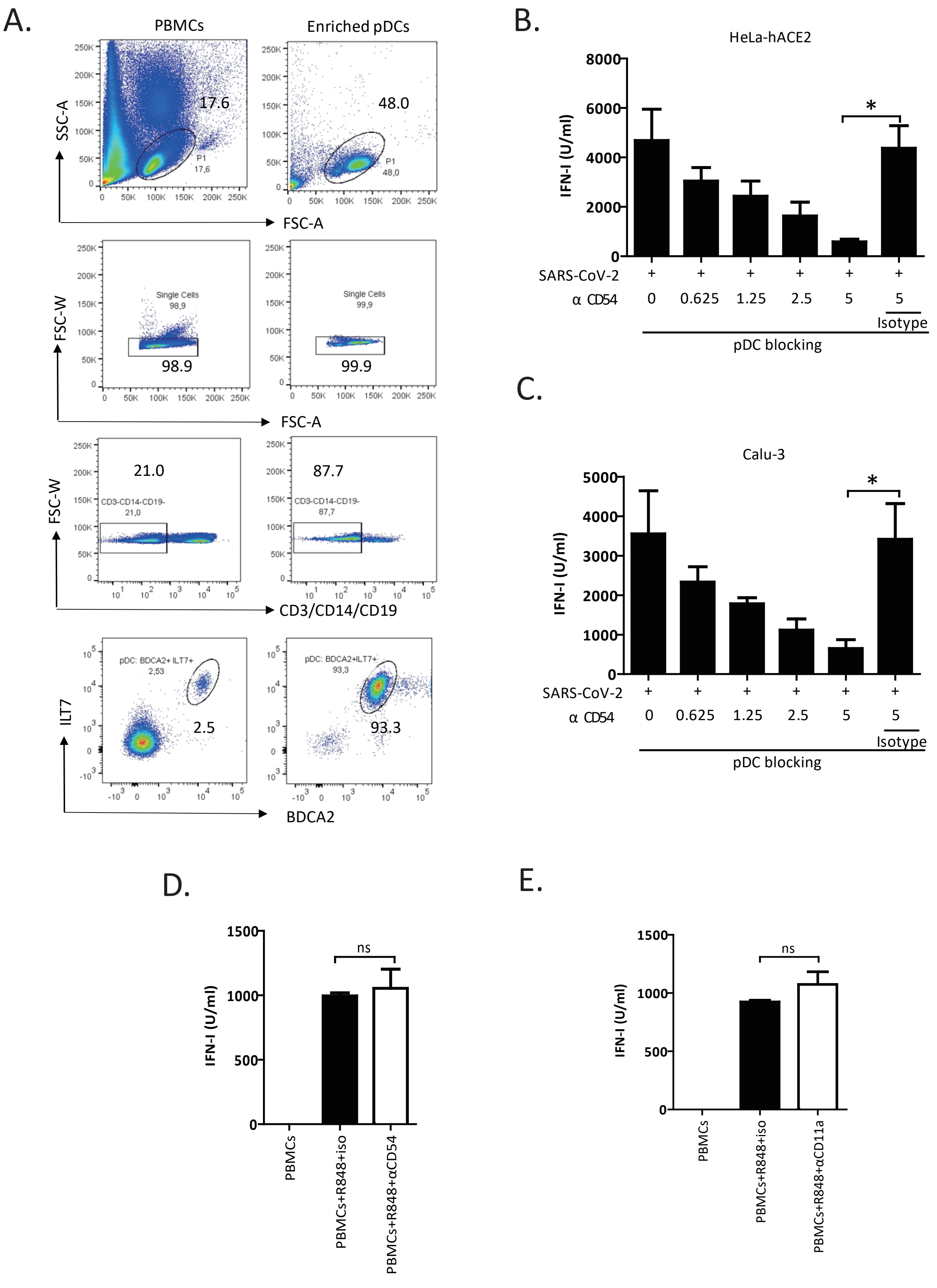

Figure S4. CD54/CD11a adhesion complex is involved in the recognition and sensing of SARS-CoV-2-infected cells by pDCs in a potentially bidirectional manner, related to Figure 5. A. Representative flow cytometry dot plots show the gating strategy to validate pDC purification by magnetic bead-based positive selection; n=5 independent analyses. The indicated markers were used to characterize the degree of pDC enrichment. **B and C.** Quantification of IFN-I release in the supernatant of infected HeLa-hACE2 or Calu-3 that were in co-culture with enriched pDCs. The latter were pre-treated with the indicated concentrations of αCD54 Ab or 5 µg/mL isotype control. IFN-I level in the untreated condition was set at 100%. Mean ± SEM; n=4. Mann-Whitney U test; *p < 0.05. **D and E.** IFN-I measurement in the supernatant of PBMCs stimulated with the TLR-7/8 agonist R848 (10 µg/mL) in the presence of (D) αCD54 Ab (5 µg/mL), (E) αCD11a Ab (10 µg/mL) or the respective isotype controls. Mean ± SEM; n=3. Mann-Whitney U test; ns, not significant.

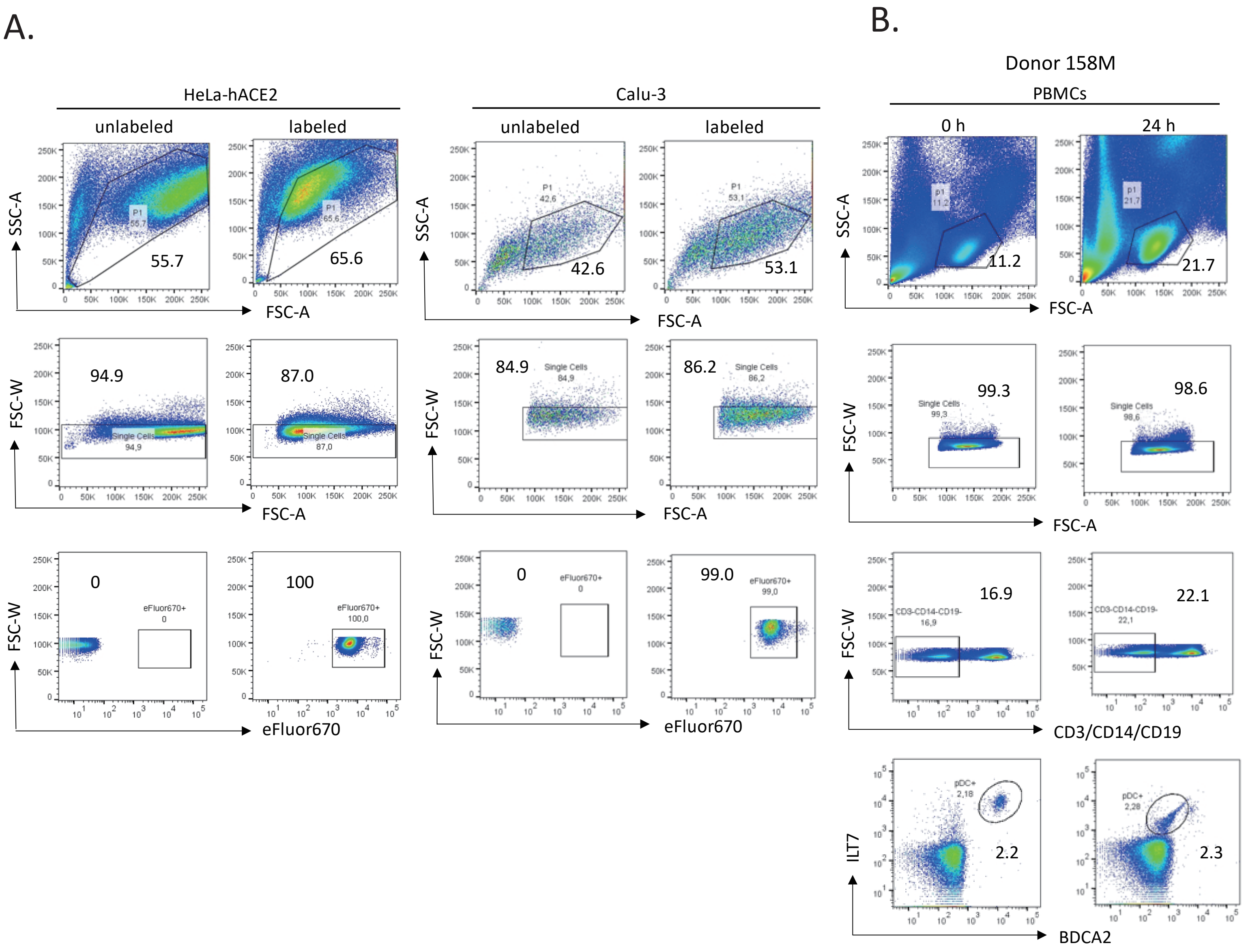

Fig S5. Effect of a 24 h culture on the level of expression of pDC-specific markers ILT7 and BDCA2 and eFluor670 staining profile for HeLa-hACE2 and Calu-3, related to Figures 6 and 7. A. HeLa-hACE2 (Left panel) or Calu-3 (Right panel) were left unlabeled or labeled with eFluor670 for 10 min at 37 ^{o}C. Binding of the dye to cells was quenched by washing cells with media supplemented with 10% FBS. Binding efficiency was verified by flow cytometry. The latter shows that all cells were labeled with eFluor670 under these conditions. **B.** Freshly isolated PBMCs (0 h) or PBMCs that were in culture for 24 h (24 h) from the same healthy donor were stained with a cocktail of fluorescently-labeled Abs and analyzed for pDC frequency by flow cytometry. PDCs were defined as $CD3^{-}CD14^{-}CD19^{-}BDCA2^{+}ILT7^{+}$. Note the decrease in the fluorescent intensity of ILT7 and BDCA2 at the 24 h time point compared to the 0 h.

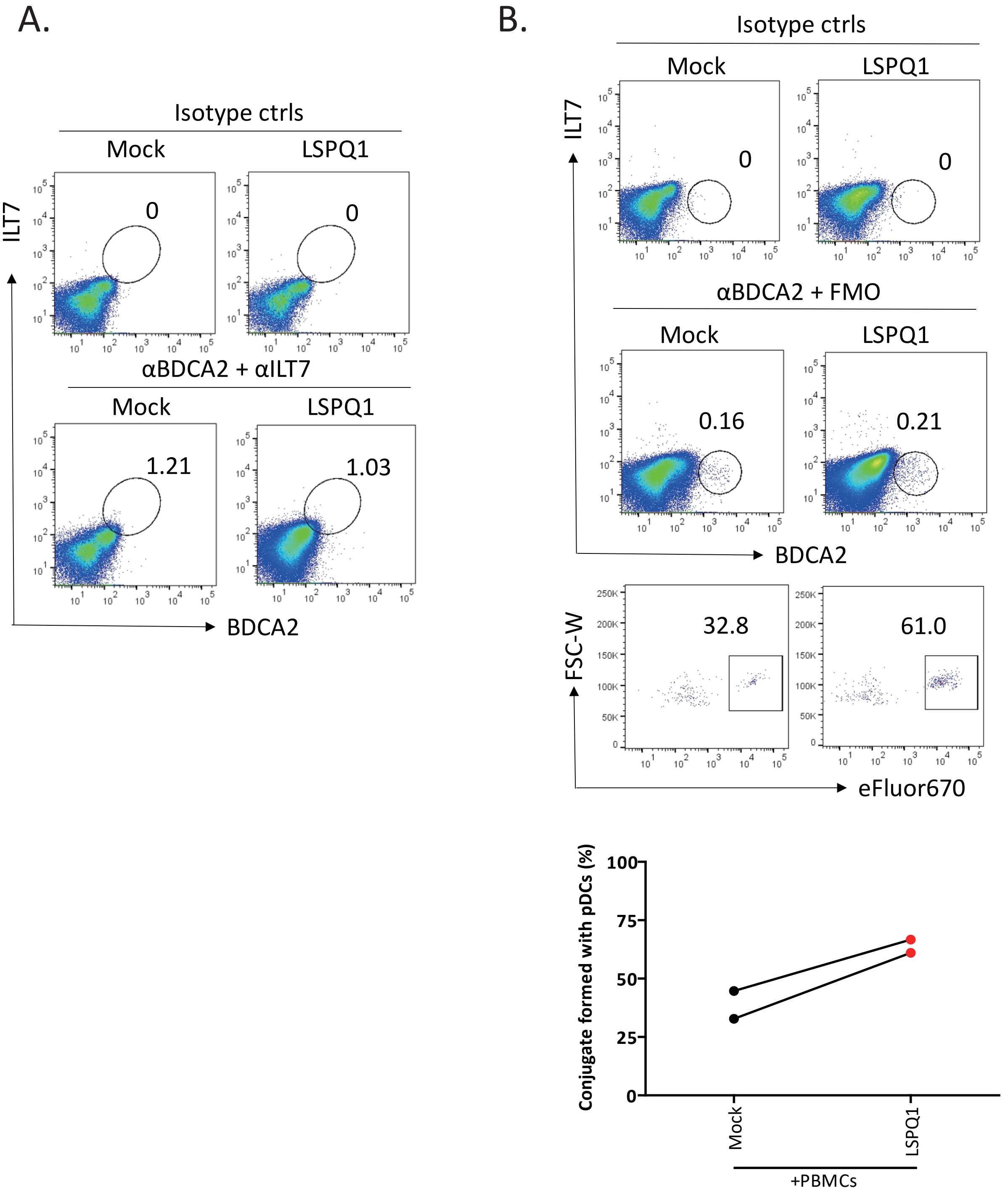

Fig S6. HeLa-hACE2 conjugate formation with pDCs, related to Figures 6 and S5. HeLa-hACE2 were infected with LSPQ1 for 24 h, followed by labeling with proliferation dye eFluor670 (1/1000) at 37°C for 10 min. Then, eFluor670-labeled HeLa-hACE2 cells were co-cultured with freshly isolated PBMCs for 24 h. By the time of determining conjugate formation, HeLa-hACE2 were infected for 48 h. **A.** Cells were stained with BV421-conjugated anti-BDCA2 (αBDCA2) and PE-conjugated anti-ILT7 (αILT7) Abs in the presence of αCD3, αCD14, and αCD19 Abs. BV421-conjugated mouse IgG2a and PE-conjugated mouse IgG1 were used as isotype controls (ctrls) for αBDCA2 and αILT7 Abs, respectively. Shown is the frequency of $CD3^-CD14^-CD19^-BDCA2^+ILT7^+$ pDCs on the basis of gating with both isotype controls. **B.** Alternatively, HeLa-hACE2 were stained with αBDCA2 Ab in the presence of αCD3, αCD14, and αCD19 Abs. The PE-conjugated mouse IgG1 was used as the fluorescence minus one (FMO) control for the staining. Depicted on the second row of Panel B is the frequency of $CD3^-CD14^-CD19^-BDCA2^+$ pDCs on the basis of the FMO gating. From there, the proportion of conjugates formed between pDCs and eFluor670-labeled HeLa-hACE2 was determined (third row). The line graph illustrates accumulated data from two independent donors (n=2, each line represents a donor).

A.

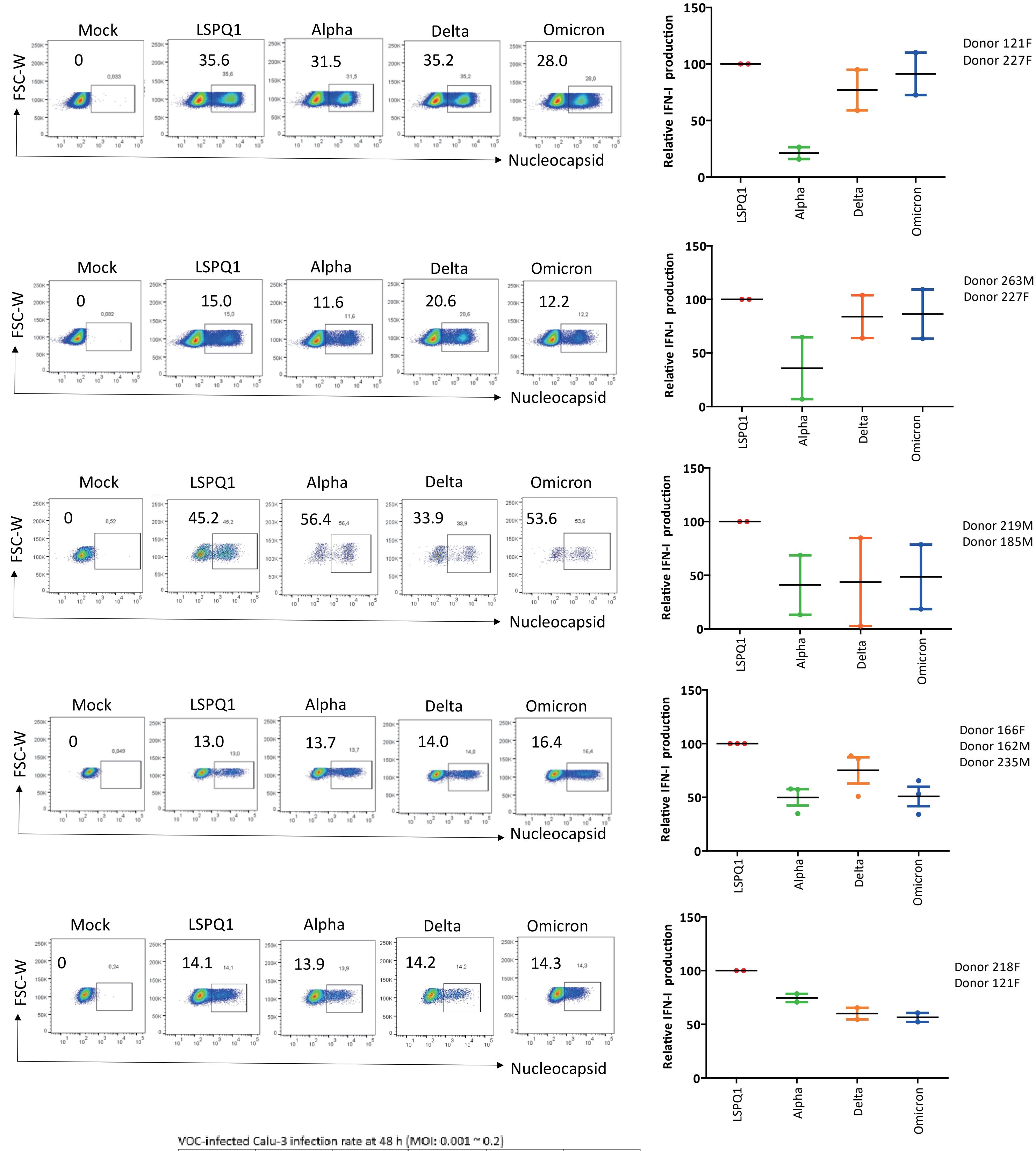

B.

VOC-infected Calu-3 infection rate at 48 h (MOI: 0.001 ~ 0.2)

	Mock	LSPQ1	Alpha	Delta	Omicron
Expt 1	0.8	67.8	64.7	74.8	57.2
Expt 2	0.6	35.0	51.6	39.0	36.7
Expt 3	0	68.3	73.6	70.4	63.7
Expt 4	0	25.8	56.8	50.7	34.0
Expt 5	0	70.6	76.1	64.1	48.0

Fig S7. IFN-I release upon sensing of VOC-infected Calu-3 by PBMCs, related to Figure 7A. Data shown in Fig. 7A are segregated by individual experiments (n=5). Calu-3 were infected with different VOCs at different MOIs (0.001 to 0.2) for up to 48 h. Infection rates were determined by flow cytometry using α-SARS-CoV-2 N Ab. **A**. The numbers shown on the flow graphs depict the percentage of infected cells at 24 h. Calu-3 with similar infection rates among the VOCs at 24 h post-infection were co-cultured with PBMCs for 24 h, and the supernatant from the co-cultures was measured for IFN-I. IFN-I production detected from sensing of LSQP1-infected Calu-3 was set at 100%. For each individual experiment, representative dot plots depicting similar infection rates among the VOCs at the beginning of the co-cultures are shown on the left, and the IFN-I production is shown on the right side. **B.** Shown is the frequency of infected cells at 48 h post infection.

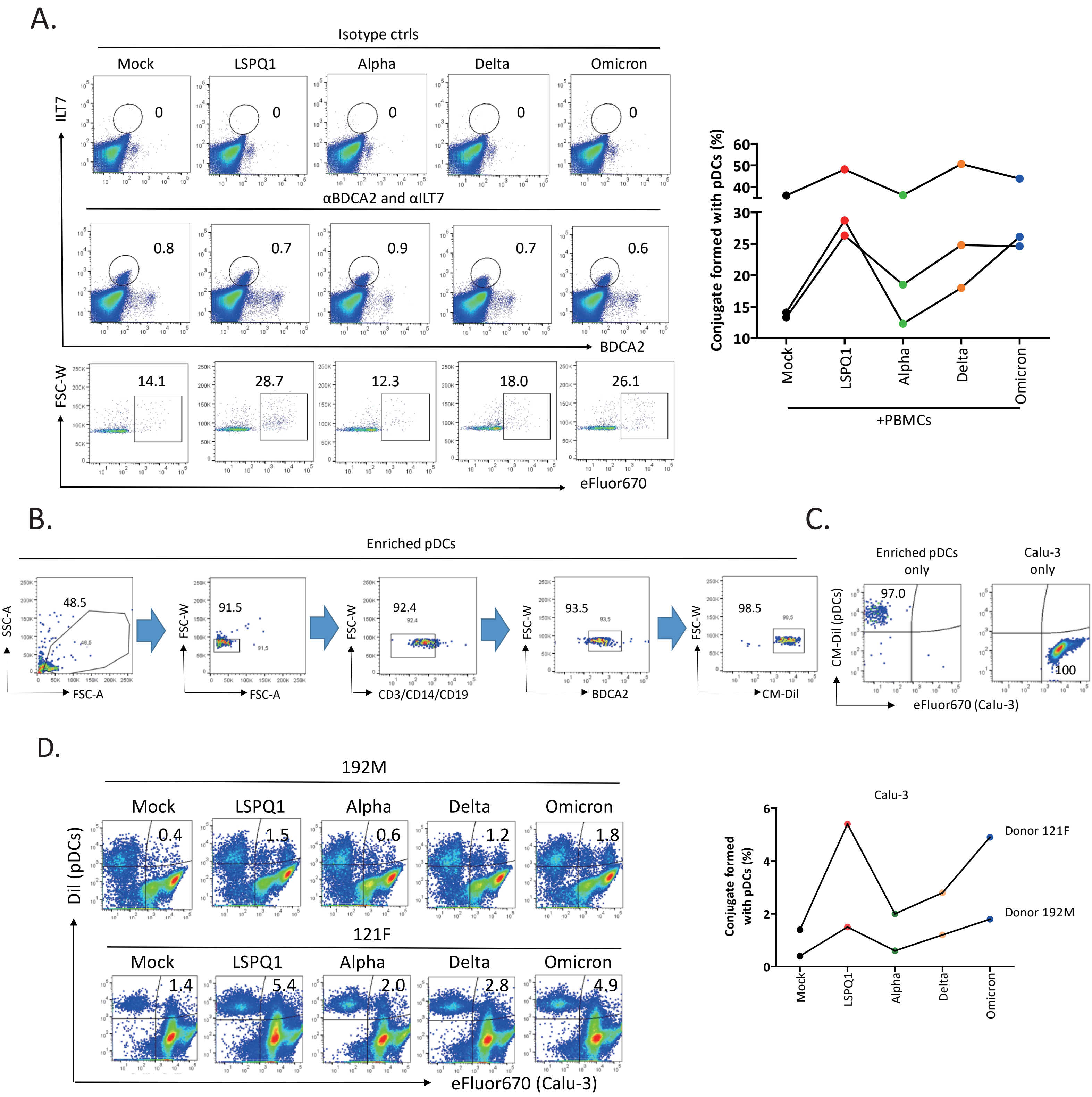

Fig S8. Isotype control for conjugate formation and conjugate formation validated with isolated pDCs in Calu-3, related to Figure 7. A. (Left panel) Shown is an experimental representative of conjugate formation of pDCs with Calu-3. Calu-3 were infected with VOCs for 24 h, followed by labelling with proliferation dye eFluor670 (1/1000) at 37 °C for 10 min. eFluor™ 670-labeled Calu-3 were then co-cultured with freshly isolated PBMCs for another 24 h. By the time of determining conjugate formation, Calu-3 were infected for 48 h. pDCs were gated from using BV421-conjugated anti-BDCA2 (αBDCA2) and PE-conjugated anti-ILT7 (αILT7) Abs in the presence of αCD3, αCD14 and αCD19 Abs. BV421-conjugated mouse IgG2a and PE-conjugated mouse IgG1 were used as isotype controls (ctrls) for αBDCA2 and αILT7, respectively. (Right panel) Accumulated data of conjugate formation between pDCs and VOC-infected Calu-3, each line represents an independent experiment, n=3. **B-C.** Representative dot plots indicate that isolated pDCs were all labelled with cell dye CM-Dil (B-C) while Calu-3 was labelled with eFluor™ 670 (C). **D.** eFluor™ 670-labeled Calu-3 was co-cultured with CM-Dil labelled pDCs for up to 24 h. At the time of the determining conjugate formation, Calu-3 cells were left uninfected (Mock) or infected with different VOCs for 48 h. Dot plots show conjugate formation between CM-Dil$^+$ pDCs and eFluor™ 670-labeled Calu-3. Numbers on the quadrant plots indicate the frequency of pDCs forming cell conjugates with Calu-3; n=2 distinct PBMC donors.

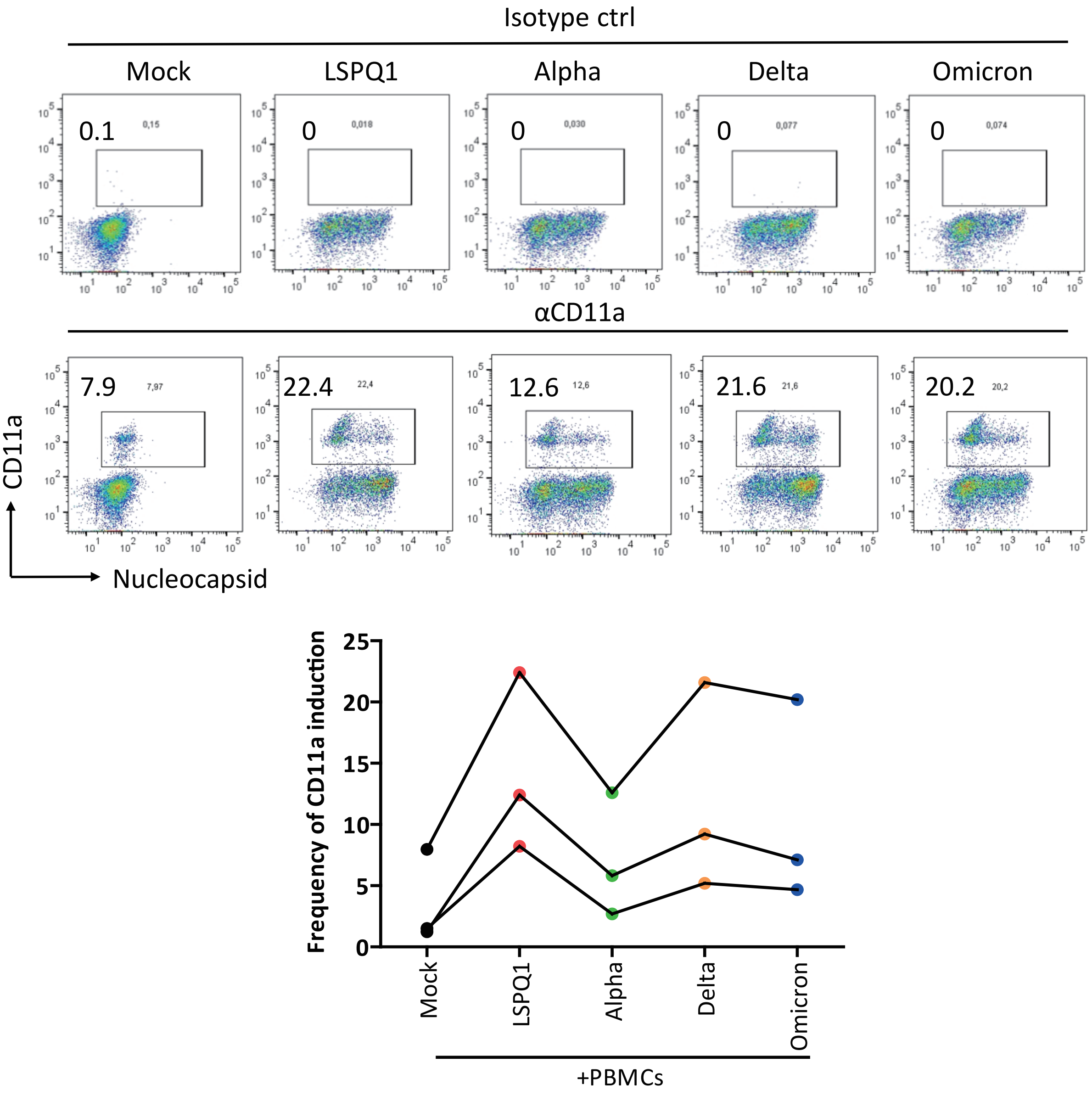

Fig S9. Isotype control for CD11a induction in Calu-3, related to Figure 7. (Upper panel) A representative of CD11a induction on Calu-3 after gating out PBMCs. Calu-3 were infected with VOCs for 24 h, followed by co-culturing with freshly isolated PBMCs for another 24 h. By the time of CD11a determination, Calu-3 were infected for 48 h. CD11a induction analysis was done in the presence of anti-CD11a Ab (αCD11a) and Abs specific for different cell subsets within PBMCs (αCD3, αCD14, αCD19, αCD11c, αHLA-DR, αBDCA2, and αILT7). PE-conjugated mouse IgG1 was used as an isotype control (ctrl). (Lower panel) Accumulated data of CD11a induction on VOCs-infected Calu-3, each line represents an independent experiment, n=3.
